# Li_6_SiO_4_Cl_2_: A Hexagonal Argyrodite Based on Antiperovskite Layer Stacking

**DOI:** 10.1021/acs.chemmater.1c00157

**Published:** 2021-03-02

**Authors:** Alexandra Morscher, Matthew S. Dyer, Benjamin B. Duff, Guopeng Han, Jacinthe Gamon, Luke M. Daniels, Yun Dang, T. Wesley Surta, Craig M. Robertson, Frédéric Blanc, John B. Claridge, Matthew J. Rosseinsky

**Affiliations:** † Department of Chemistry, University of Liverpool, Crown Street, L69 7ZD Liverpool, U.K.; ‡ Stephenson Institute for Renewable Energy, University of Liverpool, Peach Street, L69 7ZF Liverpool, U.K.

## Abstract

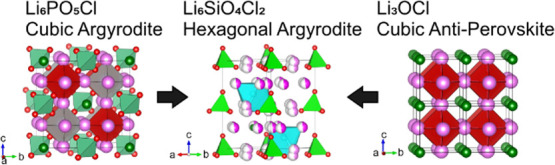

A hexagonal analogue, Li_6_SiO_4_Cl_2_, of the cubic lithium argyrodite family of solid electrolytes is isolated by a computation–experiment approach. We show that the argyrodite structure is equivalent to the cubic antiperovskite solid electrolyte structure through anion site and vacancy ordering within a cubic stacking of two close-packed layers. Construction of models that assemble these layers with the combination of hexagonal and cubic stacking motifs, both well known in the large family of perovskite structural variants, followed by energy minimization identifies Li_6_SiO_4_Cl_2_ as a stable candidate composition. Synthesis and structure determination demonstrate that the material adopts the predicted lithium site-ordered structure with a low lithium conductivity of ∼10^–10^ S cm^–1^ at room temperature and the predicted hexagonal argyrodite structure above an order–disorder transition at 469.3(1) K. This transition establishes dynamic Li site disorder analogous to that of cubic argyrodite solid electrolytes in hexagonal argyrodite Li_6_SiO_4_Cl_2_ and increases Li-ion mobility observed via NMR and AC impedance spectroscopy. The compositional flexibility of both argyrodite and perovskite alongside this newly established structural connection, which enables the use of hexagonal and cubic stacking motifs, identifies a wealth of unexplored chemistry significant to the field of solid electrolytes.

## 1 Introduction

Compounds related to the mineral argyrodite (Ag_8_GeS_6_) have been the focus of considerable interest for over half a century. The argyrodite family is compositionally flexible, with the general formula A_(12–*n*–*y*)/*m*_^*m*+^L_6–*y*_^*n*+^X_6–*y*_^2–^Y_*y*_^–^ (A = Ag^+^, Cu^+^, Cd^2+^, etc.; L = Ga^3+^, Si^4+^, Ge^4+^, P^5+^, etc.; X = S^2–^, Se^2–^, Te^2–^; Y = Cl^–^, Br^–^, I^–^),^1^ where the A cation content can vary from 3.25 to 9 to maintain charge neutrality. This family of compounds contains examples of fast Ag^+^ and Cu^+^ ion conductors (e.g., Cu_6_PS_5_Cl^1^ and Ag_7_NbS_6_^2^) as well as materials interesting for their nonlinear optical properties (e.g., Cd_3.25_PS_5.5_I_0.5_^1^) and thermoelectric properties (e.g., Ag_8_SnSe_6_^3^).

The reported crystal structures of argyrodites are related to the high-temperature *F*4̅3*m* cubic polymorph of Ag_8_GeS_6_, where A cations often demonstrate an extended dynamic disorder. Static ordering of cations at low temperatures, along with the corresponding structural displacements, can lead to low-temperature polymorphs with lower symmetry.^4,5^ This dynamic disorder in higher-symmetry polymorphs has led to extensive investigations of argyrodites as potential solid-state electrolytes in all solid-state lithium-ion batteries (ASSBs).^6,7^ In the lithium-containing argyrodites, the introduction of a halide anion transforms the room-temperature orthorhombic polymorph (Li_7_PCh_6_ (Ch = S, Se): space group *Pna*2_1_) into cubic symmetry (Li_6_PCh_5_X (X = Cl, Br, I): space group *F*4̅3*m*), while charge compensation occurs via reduction of the lithium content. The latter cubic phase exhibits an order–disorder phase transition from static order (LT cubic phase) to an extended dynamic disorder (HT cubic phase), with delocalization of the lithium distribution.^8^ In cubic oxide analogues such as Li_6_PO_5_Br, this phase transition does not occur, and lithium localization within the *F*4̅3*m* structure is maintained over a wide temperature range (173–873 K).^9^ In the ordered *F*4̅3*m* structure of Li_6_PO_5_Br, lithium ions fully occupy a single crystallographic 24*g* position. In the dynamically disordered HT *F*4̅3*m* structure of the lithium sulfide argyrodites, the additional available lithium sites (Wyckoff position 48*h*) and extended diffusion of Li^+^ are linked to a dramatic increase in ionic conductivity by six orders of magnitude.^10,11^ The most highly conducting Li-containing argyrodites are based on mixed sulfide–halide compositions, e.g., Li_6_PS_5_Br^6^ and Li_6.35_P_0.65_Si_0.35_S_5_Br, in which Li^+^ is delocalized across 48*h* and 24*g* positions.^12^

The high-temperature *F*4̅3*m* argyrodite structure has previously been described in terms of tetrahedral close-packing^1^ or as localized pseudo-octahedral cages of A cations surrounding one anion and separated by the other anions.^10,11^ In this work, we establish a new relationship between the structures of argyrodites and those of cubic antiperovskites, which are themselves good conductors of lithium ions (i.e., Li_3_OCl_1–*x*_Br*_x_*).^13^ We extend this relationship, which provides a framework for designing lithium-ion conductors, from cubic to hexagonal antiperovskites and thus propose the lithium hexagonal argyrodite family, computationally identifying several new compositional targets within this family. Li_6_SiO_4_Cl_2_ is then successfully synthesized following these predictions based on mixed cubic and hexagonal stacking and shows analogous dynamical cation site disorder to cubic argyrodites, which is required for Li transport.

## 2 Experimental Procedure

### 2.1 Computational Methods

Periodic plane-wave-based density functional theory (DFT) calculations were performed using the VASP code (version 5.4.4).^14^ All calculations were performed with the projector-augmented wave method,^15^ a plane-wave cutoff energy of 700 eV, and a *k*-point spacing of 0.15 Å^–1^. Geometry optimization of both atomic positions and unit cell parameters was terminated once all forces fell below 0.001 eV Å^–1^. The PBE functional^16^ was used to calculate relative energies and mechanical properties, and the PBEsol functional^17^ was used for direct comparison between computational and experimental crystal structures. The convex hull of energies for all reported phases in the Li–Si–O–Cl–Br and Li–Si–O–Cl–I phase fields was calculated using pymatgen.^18^ Normal mode calculations were performed using the harmonic approximation, with finite displacements of 0.01 Å and including distortions of the unit cell. This allowed the calculation of elastic constants, including the bulk and shear moduli.^19^ For comparison with existing compounds, the elastic constants of Li_3_OBr (*Pm*3̅*m* structure)^20^ and Li_6_PS_5_Br (*Cc* structure of Li_6_PS_5_I)^21^ were computed using the same method.

### 2.2 Synthesis

#### 2.2.1. Materials

Li_2_CO_3_ (99.99%), SiO_2_ (silica gel, technical grade, particle size 40–63 μm), and LiCl (>99.0%) were purchased from Sigma-Aldrich.

#### 2.2.2. Synthesis of Li_4_SiO_4_^22^

Precursors were dried overnight at 473 K before weighing. Li_2_CO_3_ (1.2331 g,) and SiO_2_ (0.5013 g) were weighed according to the stoichiometric 2:1 ratio. The powders were ground with an agate pestle and mortar for 15 min, placed into an alumina crucible and heated in air to 1073 K at a ramp rate of 5 K min^–1^, held at 1073 K for 12 h, and cooled at a ramp rate of 5 K min^–1^. The resulting product was ground to obtain a fine powder, which was then used as a precursor in the final synthesis step.

#### 2.2.3. Synthesis of Li_6_SiO_4_Cl_2–*x*_Br*_x_*

Li_4_SiO_4_, LiCl, and LiBr were vacuum-dried overnight (under 10^–4^ mbar) before placing them in an Ar-filled glovebox. All precursors and resulting powders were then handled in an Ar-filled glovebox. LiCl, LiBr, and Li_4_SiO_4_ were mixed in the stoichiometric ratio, ground with an agate pestle and mortar for 15 min, and transferred to an alumina crucible. The crucible was placed in a silica tube, which was sealed under vacuum (<10^–4^ mbar). The tube was heated to 798 K at a ramp rate of 5 K min^–1^, held at 798 K for 12 h, and cooled at a rate of 5 K min^–1^. Once cooled, the silica tube was opened inside the Ar glovebox, and the powder was ground in a pestle and mortar for further characterization.

Single crystals of Li_6_SiO_4_Cl_2_ were grown by mixing LiCl and Li_4_SiO_4_ in the stoichiometric ratio, heating the mixture to 883 K at a ramp rate of 5 K min^–1^, annealing it for 3 h, and slow cooling at 3 K h^–1^ to room temperature.

### 2.3 Powder X-ray Diffraction

Routine assessment of sample purity was carried out using a Bruker D8 Discover diffractometer with monochromatic Cu radiation (Kα_1_, λ = 1.54056 Å) in a Debye Scherrer transmission geometry with sample powders loaded into 0.5 mm borosilicate glass capillaries.

Synchrotron X-ray diffraction (SXRD) was performed at Diamond Light Source U.K., on high-resolution beamline I11,^23^ at λ = 0.826552 Å. The pattern was recorded in transmission mode [0° < 2θ < 150°] using a multianalyzer crystal (MAC) detector. The sample was introduced into a 1.0 mm diameter borosilicate glass capillary. The experiment was performed at room temperature.

Synchrotron variable temperature X-ray diffraction (VT-XRD) was performed in transmission mode using a position-sensitive detector (PSD, λ = 0.82660 Å) on a sample, which was introduced into a 1.0 mm diameter silica capillary. The experiment was performed in the temperature range of 298–798 K in 25 K steps on heating and then cooled directly to room temperature to assess reversibility.

Rietveld refinements were carried out using TOPAS Academic.^24^ Initially, Pawley fits were performed on SXRD data, refining the lattice parameters and the background using a Chebyshev function with 12 parameters. The peak shape was modeled using a pseudo-Voigt function (high-temperature patterns) and a Thomson–Cox function (room-temperature patterns). Refined parameters from final Pawley fits were then used as starting points for Rietveld refinements where the following parameters were refined: (1) scale factor, (2) atomic coordinates, (3) isotropic (Li, Si, O) and anisotropic (Cl) displacement parameters, and (4) atomic occupancies: the occupancies of Cl and O, and Li were refined, while the occupancy of the Si site was set to the nominal value and the Li occupancies were set to ensure charge compensation.

### 2.4 Single-Crystal X-ray Diffraction

A twinned crystal was examined with a Rigaku MicroMax-007 HF X-ray generator equipped with a Mo Kα rotating-anode microfocus source and a Saturn 724+ detector. The data were collected at 100 K. Refinement of the cell parameters, indexing of twin components, and reduction of the data were performed on the obtained diffraction images with the use of the software package CrysAlisPro.^25^ The volume ratio of the twin components was about 1:1. The degree of overlap of two components is around 1%. The twin operator is 92.82° rotation around the −0.59 0.37 −0.72 reciprocal axis. The detwinned HKLF4 format file was used for structure solution using Olex2.^26^ The crystal structure was solved with the intrinsic phasing method provided by the ShelXT^27^ structure solution program. Refinements were carried out with the ShelXL^28^ refinement package using least-squares minimization.

### 2.5 Differential Scanning Calorimetry (DSC)

Heat flux profiles were measured from 15.1 mg of powdered sample in a 40 μL aluminum crucible cold-welded under an Ar atmosphere (<0.1 ppm O_2_, H_2_O) using a Netzsch DSC 404 F1 differential scanning calorimeter. Data were recorded on heating to 823 K and then cooling to 323 K using heating and cooling rates of 10 K min^–1^ under a constant 50 mL min^–1^ flow of helium. The transition temperature is the average of the values obtained from both the heating and cooling curves, which were extracted through peak fitting.

### 2.6 Alternating current (AC) Impedance Spectroscopy and Direct Current (DC) Polarization

A pellet of Li_6_SiO_4_Cl_2_ was made by uniaxially pressing ∼30 mg of material in an 8 mm cylindrical steel die at a pressure of 125 MPa. The pellet was sintered in an evacuated, flame-dried silica tube for 12 h at 848 K. Using this method, a relative density of 84% was achieved.

AC impedance measurements were conducted using an impedance analyzer (Keysight impedance analyzer E4990A). A sputtered gold coating of 300 nm thickness was used as the ion blocking electrodes. Sputtering was achieved under an argon atmosphere using a Q150R sputter coater, and temperature-dependent conductivity measurements were performed under an argon atmosphere over a frequency range of 2 MHz to 20 Hz (with an amplitude of 100 mV). Measurements were performed in the temperature range of 333–575 K in 20 K steps. The ZView2 program^29^ was used to fit the impedance spectra with an equivalent circuit.

A pellet of 84% relative density was used for DC potentiation polarization measurements. DC polarization data was collected at 300 °C on a Au|Li_6_SiO_4_Cl_2_|Au symmetric cell by applying constant potentials of 0.05, 0.1, 0.5, and 1 V for 7200 s and monitoring the current variation with time. Once a constant current was achieved, the current was recorded and plotted against the applied voltage allowing for the electronic conductivity (σ_e_) to be extracted from the following equation
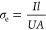
1where *U* refers to the polarization voltage, *l* refers to the pellet thickness, *A* refers to the Au electrode area, and *I* refers to the current.

### 2.7 Nuclear Magnetic Resonance (NMR) Spectroscopy

^29^Si magic-angle spinning (MAS) NMR spectra were recorded with a 4 mm HXY MAS probe in double resonance mode on a Bruker 9.4 T Avance III HD spectrometer. ^29^Si NMR data was obtained using a pulse length of 5 μs at a radio frequency (rf) amplitude of 50 kHz and at an MAS rate of ν_r_ = 10 kHz. The sample was packed into a rotor in an Ar-filled glovebox to eliminate exposure to air and moisture. ^29^Si chemical shifts were externally referenced to the lowest-frequency signal of octakis(trimethylsiloxy)silsesquioxane at −109 ppm,^30^ relative to tetramethylsilane primary reference at 0.0 ppm.

Variable temperature ^7^Li NMR experiments were recorded with a 4 mm HX High-Temperature MAS Probe on a 9.4 T Bruker Avance III HD spectrometer under static conditions with the X channel tuned to ^7^Li at ω_0_/2π (^7^Li) = 156 MHz. The sample was sealed in a glass ampoule, and the spectra were recorded with a pulse length of 1.5 μs at an rf field amplitude of ω_1_/2π = 83 kHz and referenced to 10 M LiCl in D_2_O at 0 ppm.

The homonuclear dipolar coupling constant between two ^7^Li nuclear spins *d*_7Li7Li_ (in Hz) can be calculated via the following expression
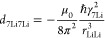
2where μ_0_ is the permeability of free space, ℏ is the reduced Planck’s constant, γ_7Li_ is the gyromagnetic ratio of ^7^Li, and *r_LiLi_* is the Li–Li distance.

Temperature calibrations were performed using the chemical shift thermometers Pb(NO_3_)_2_ using ^207^Pb NMR^31^ and by monitoring the phase transition of CuI and CuBr using ^63^Cu NMR.^32,33^ The errors associated with this method were calculated using the isotropic peak line broadening and range from 5 to 20 K.

### 2.8 Maximum Entropy Methods (MEM)

Maximum entropy method (MEM) analysis was performed on SXRD and VT-XRD data using the software Jana2006^34^ and BayMEM.^35^ Rietveld models from Topas were input into Jana2006, which was used to extract *F*_obs_ and generate MEM inputs. BayMEM was used to calculate the electron density distribution using the *F*_obs_ and the number of electrons in the nominal stoichiometry. Calculations using the number of electrons associated with a 10% Li deficiency were also performed for all models investigated and found to have negligible impact in the resulting electron density distributions. All MEM results were visualized in the VESTA crystal structure visualization software.^36^

## 3 Results and Discussion

### 3.1 Selection of Target Compounds by Structural Analogy between Argyrodite and Perovskite

**Figure 1:**
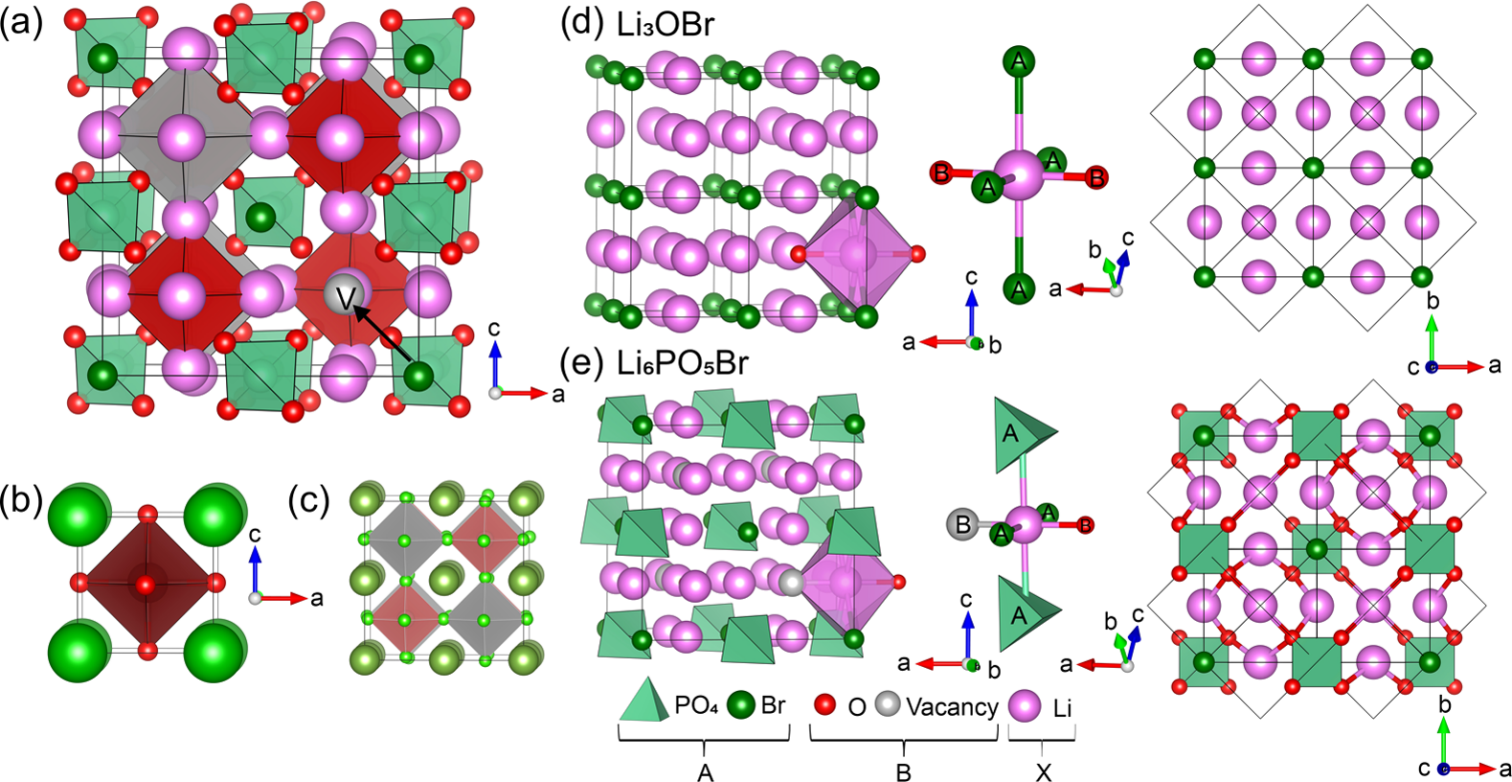
(a) Cubic argyrodite structure of Li_6_PO_5_Br (Li: pink, P: light green, O: red, Br: dark green, vacant B-site (V): gray; the arrow shows the direction of the P–O bond vector oriented toward the vacant B-site), showing the relationship with (b) the cubic perovskite SrTiO_3_ (Sr: green, Ti: dark red, O: red) and (c) the B-site vacancy-ordered K_2_PtCl_6_ (K: dark green, Pt: red, Cl: light green, vacancies: gray). (d) Cubic antiperovskite structure of Li_3_OBr, highlighting the octahedral coordination environment of Li, coordinated by four A-site (Br: dark green) and two B-site (O: red) anions. (e) Compared with Li_3_OBr, the cubic argyrodite structure of Li_6_PO_5_Br has a reduced Li coordination number defined by four A-site (Br: dark green, PO_4_: polyanion light green) and one B-site anion (O: red) with half of the B-sites vacant (vacancy: gray).

We describe the relationship between the argyrodite structure, exemplified by Li_6_PO_5_Br (Figure 1a), and the cubic antiperovskite structure. In analogy with perovskites of general formula ABX_3_, Li_6_PO_5_Br can be written as [((PO_4_)_0.5_Br_0.5_)(O_0.5_□_0.5_)Li_3_]_2_, where □ is a vacancy. As such, Li_6_PO_5_Br could be considered as an inverse double perovskite. When viewed as a cubic antiperovskite, the lithium ions in Li_6_PO_5_Br replace the anion positions in the conventional cubic perovskite (e.g., SrTiO_3_;^37^Figure 1b). The phosphate polyanions and bromide anions replace the larger A-site cations in a rock-salt-ordered fashion. As the cubic perovskite can be represented as a cubic close-packed stacking of AX_3_ layers along [111], here the cubic stacking is an alternation of Li_3_Br and Li_3_PO_4_ layers that produces the rock-salt anion ordering. The remaining, isolated oxide anions, then occupy half of the octahedral B-site positions between these layers also in an ordered manner, leaving the other half of the B-sites vacant. The P–O bond vector of each tetrahedral (PO_4_)^3–^ anion is directed toward the vacant B-sites, coupling the rock-salt order of the two A-site anions to the anion vacancy defect ordering on the B-site. Rock-salt order of 50% B-site vacancies occurs in the structure of well-known K_2_PtCl_6_^38^ (Figure 1c). When considering these structural similarities between the lithium argyrodites and the lithium antiperovskites, it is evident that the lithium sites in both structures are topologically equivalent. As a result, both structures share the same network of connectivity between lithium sites (Figure 1d,e), suggesting that the underlying mechanism for high ionic conductivity is common to both material families. The existence of both cubic and hexagonal perovskites associated with the distinct stackings of the AX_3_ layers suggests the existence of hexagonal Li argyrodites that are also based on alternating Li_3_Br and Li_3_PO_4_ stackings.

Although closely related in composition to argyrodite, the compound Cu_8_GeSe_6_ has a hexagonal *P*6_3_*mc* high-temperature polymorph rather than the cubic *F*4̅3*m* argyrodite structure,^39−41^ as do the compounds Ag_5_PS_4_I_2_^42^ and Li_8_SiO_6_.^43^^43^ Analysis of these structures shows that they contain similar close-packed layers to those present in the cubic antiperovskite description of the argyrodite family, but that, instead of a −c– (−a–b–c−) close-packed stacking, they are stacked in a −h–c–h–c– (−a–b–a–c−) manner, as would occur in a hexagonal antiperovskite. Different close-packed layer stacking motifs are known within the antiperovskites.^44^ The argyrodite/cubic antiperovskite structural relationship suggests that Li-containing materials based on the hexagonal, rather than cubic, antiperovskite stacking of these layers, related to the hexagonal structures of Cu_8_GeSe_6_ and Ag_5_PS_4_I_2_, would be analogous to the lithium-containing argyrodites, where the number of Li sites and extent of Li ordering among them control ionic conductivity.

**Figure 2:**
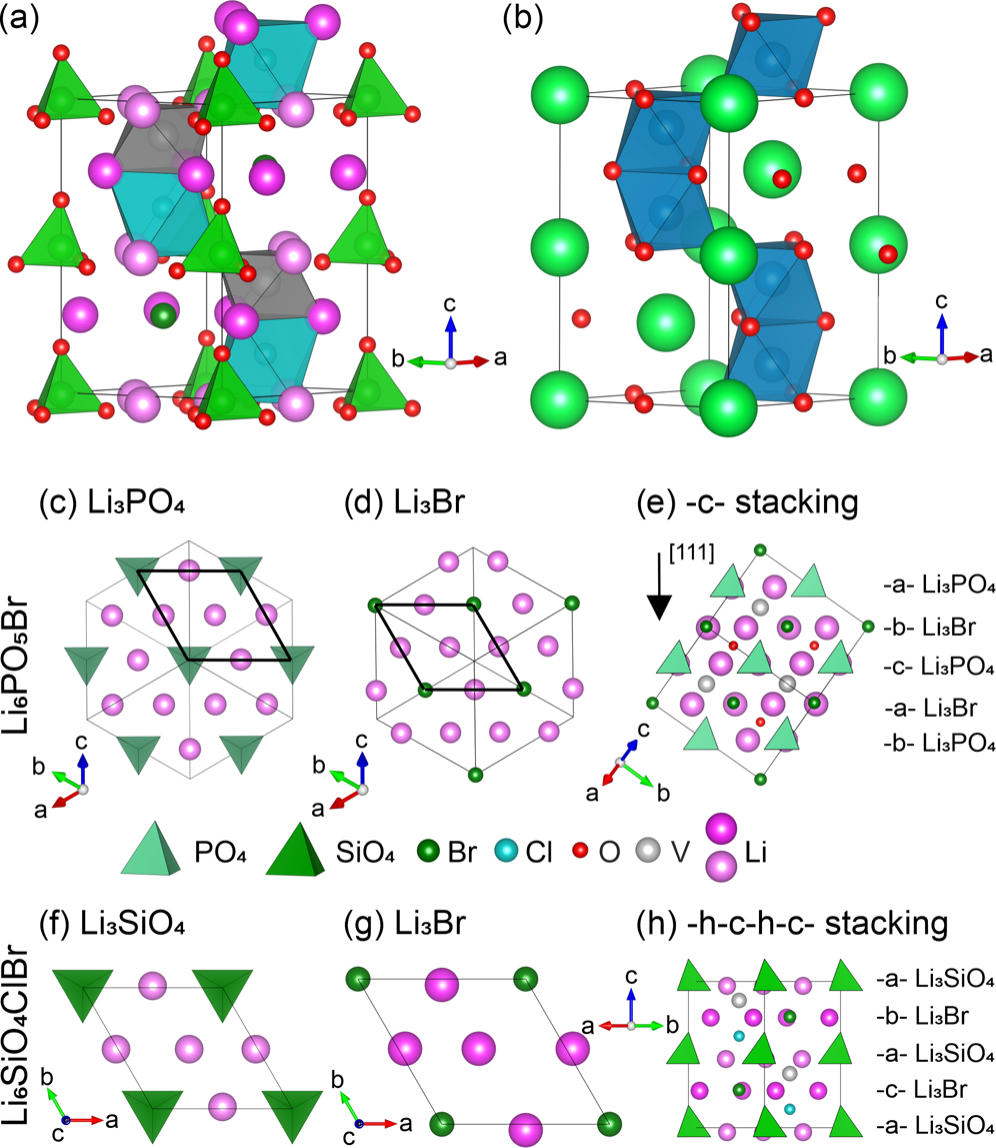
(a) DFT-optimized *P*6_3_*mc* structure of Li_6_SiO_4_ClBr (Li: pink, Si: light green, O: red, Br: dark green, Cl: blue, vacant B-site: gray) is compared to (b) the 4H-BaMnO_3_ hexagonal perovskite structure (Ba: green, Mn: blue, O: red). The oxide anions in the conventional hexagonal perovskite are replaced by lithium cations in Li_6_SiO_4_ClBr, the A-site barium cations are replaced by an ordered mixture of silicate polyanions and bromide anions, and the B-site manganese cations are replaced by ordered chloride anions and vacancies. In Li_6_SiO_4_ClBr, close-packed Li_3_Br layers (dark pink lithium) alternate along the *c*-direction with close-packed Li_3_SiO_4_ layers (light pink lithium) in an –a–b–a–c– stacking sequence. Half of the octahedral B-sites between these layers are then occupied by chlorine anions (blue octahedra), leaving half of the B-sites vacant (gray octahedra). (c–h) Comparing close-packed AX_3_ layers in Li_6_PO_5_Br cubic argyrodite (viewed along the [111] direction in (c) and (d), and computed Li_6_SiO_4_ClBr hexagonal argyrodite viewed along [001] in (f) and (g)): (c) Li_6_PO_5_Br: Li_3_PO_4_ layer, (d) Li_6_PO_5_Br: Li_3_Br layer, and (e) Li_6_PO_5_Br: cubic (−a–b–c−) stacking of close-packed Li_3_Br and Li_3_PO_4_ layers. Oxygen atoms (red) and vacancies (gray) occupy octahedral B-sites between the close-packed layers (f) Li_6_SiO_4_ClBr: Li_3_SiO_4_ layer, (g) Li_6_SiO_4_ClBr: Li_3_Br layer, and (h) Li_6_SiO_4_ClBr: −h–c–h–c– (−a–b–a–c–) stacking of close-packed Li_3_Br and Li_3_SiO_4_ layers. Chlorine atoms (blue) and vacancies (gray) occupy octahedral B-sites between the close-packed layers. For all further figures, all (poly)anions occupying the A-site are drawn in green and all B-site anions are drawn in blue.

Starting from the cubic Li_6_PO_5_X (X = Cl, Br) argyrodites, the phosphate (PO_4_)^3–^ (P–O bond length: 1.54 Å) was replaced with silicate (SiO_4_)^4–^ (Si–O bond length: 1.65 Å) to increase the A-site cation radius and thus drive a cubic to hexagonal transition through the perovskite tolerance factor (*r*_P(V)_ = 0.17 Å, *r*_Si(IV)_ = 0.26 Å).^45^ The isolated oxide ion was replaced by a halide ion (F, Cl, Br, I) to balance charge. Structures were built with compositions Li_6_SiO_4_XX′ (X, X′ = F, Cl, Br, I) with the –h–c–h–c– (–a–b–a–c−) stacking pattern, following Cu_8_GeS_6_, in the *P*6_3_*mc* space group. This affords a hexagonal antiperovskite in which half of the A-sites are occupied with silicate polyanions and half with halide anions. Half of the B-sites are then occupied in an ordered manner by the remaining halide anions, with the vacant B-sites chosen to avoid interactions between the halide anions and the corners of the silicate tetrahedra (Figure 2a).

This is a hexagonal analogue of the Li_6_PO_5_Br structure described previously and an inverse analogue of the 4H-hexagonal perovskites (e.g., 4H-BaMnO_3_,^46^ stacking sequence –h–c–h–c (–a–b–a–c–); Figure 2b). These hexagonal Li argyrodites contain similar close-packed AX_3_ layers to the cubic argyrodites (i.e., alternating Li_3_Br and Li_3_PO_4_/Li_3_SiO_4_ layers) but they are stacked in a –h–c–h–c– (–a–b–a–c−) pattern and not a −c− (–a–b–c−) stacking pattern (Figure 2c–h). These compositions were then screened computationally to find the lower-energy structures.

**Figure 3:**
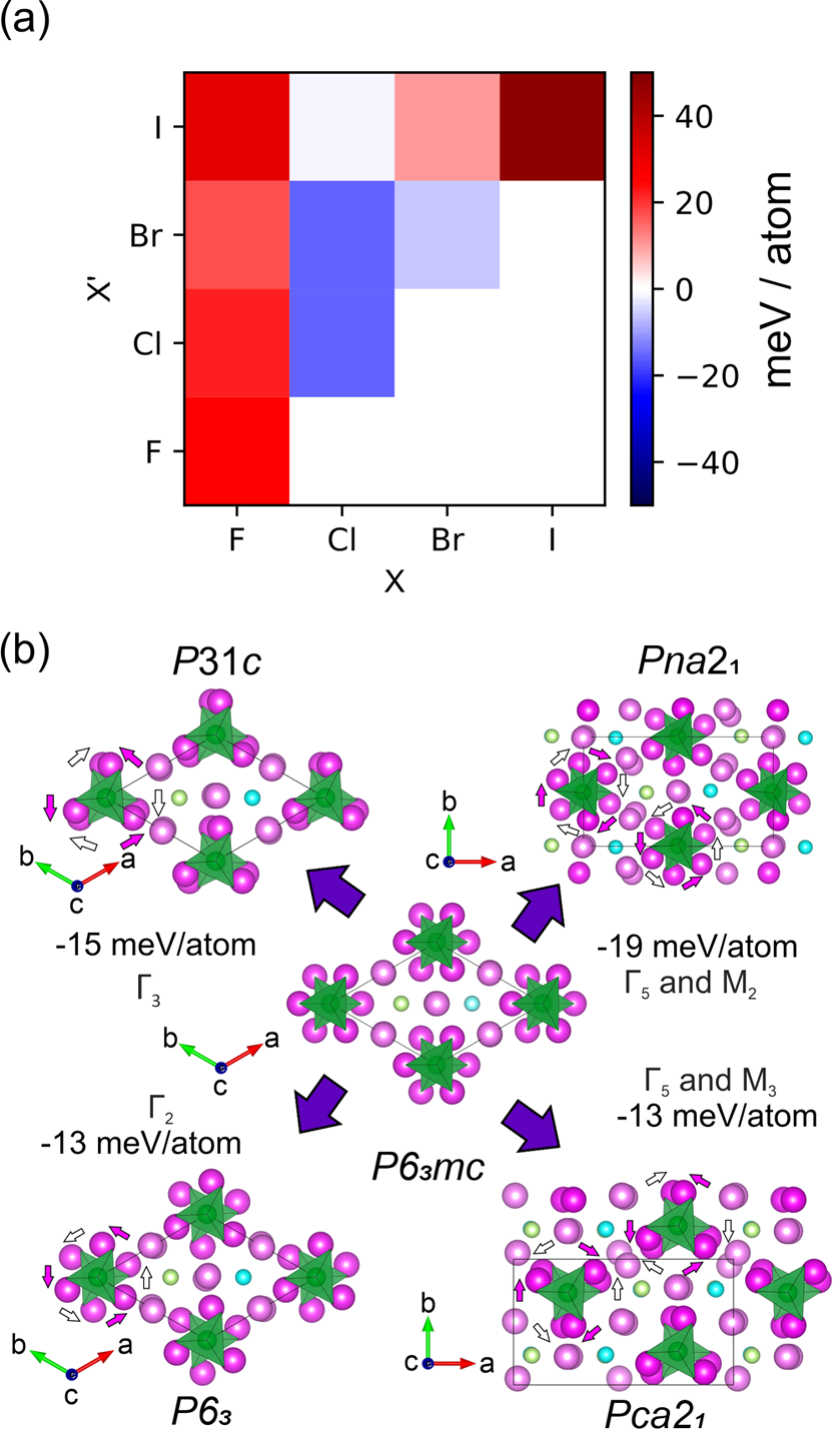
(a) Stabilities of the compounds Li_6_SiO_4_XX′ (X/X′ = F^–^, Cl^–^, Br^–^, I^–^) against decomposition into Li_4_SiO_4_ + LiX + LiX′, calculated using DFT. Li_6_SiO_4_BrCl and Li_6_SiO_4_Cl_2_ are calculated to be the most stable compositions. (b) DFT-optimized structures of Li_6_SiO_4_Cl_2_ (Li: pink, SiO_4_: green, Cl: light green and blue). In the high-symmetry *P*6_3_*mc* structure (center), half of the lithium ions lie on mirror planes (shown in darker pink). Displacement of these lithium ions off the mirror planes leads to a lowering of symmetry and a reduction in the DFT-calculated energy. Triangles of lithium ions are displaced by small rotations about the *c* axis. These rotations can be in phase or out of phase, leading to the different low-symmetry structures shown. Rotations of lithium ions in the Li_3_SiO_4_ layers and Li_3_Cl layers are shown by pink and white arrows, respectively.

Periodic DFT calculations showed that the composition Li_6_SiO_4_ClBr in this *P*6_3_*mc* structure was the most stable when compared with a stoichiometric combination of lithium orthosilicate Li_4_SiO_4_ and the relevant binary lithium halides (LiCl and LiBr) (Table S1). Through phonon calculations, lower-energy structures than the high-symmetry hexagonal structures were found, arising from the rotation of lithium atoms off the mirror plane of *P*6_3_*mc* through activation of displacive Γ_2_, Γ_3_, Γ_5_, M_2_, and M_3_ modes (Figure 3b), leading to four potential lower-symmetry polymorphs in space groups *P*31*c*, *P*6_3_, *Pna*2_1_, and *Pca*2_1_. The stabilities of all compositions were recomputed in these four lower-symmetry polymorphs, revealing that Li_6_SiO_4_Cl_2_, Li_6_SiO_4_Br_2_, Li_6_SiO_4_ClBr, and Li_6_SiO_4_ClI were predicted to be stable (Figure 3a, Table 1, and Table S1).

**Table 1:** Computed Decomposition Energies of the Compounds Li_6_SiO_4_XX′ in the Most Stable Symmetry

composition	energy meV/atom	symmetry
Li_6_SiO_4_Cl_2_	–18	*Pna*2_1_
Li_6_SiO_4_ClBr	–16	*Pna*2_1_
Li_6_SiO_4_Br_2_	–6	*Pna*2_1_
Li_6_SiO_4_ClI	–2	*P*6_3_*mc*

These results suggest that a new lithium hexagonal argyrodite family of compounds may be experimentally accessible, with Li_6_SiO_4_Cl_2_ and Li_6_SiO_4_ClBr (with the larger Br ordered on the A-site) predicted to be the most stable and chosen as targets for experimental synthesis.

DFT calculations of the elastic constants of Li_6_SiO_4_Cl_2_ in the *Pna*2_1_ structure result in a computed bulk modulus, *B*_DFT_, of 53 GPa and a computed shear modulus, *G*_DFT_, of 31 GPa. This material is considerably softer than oxide solid-state lithium electrolytes such as Li_7_La_3_Zr_2_O_12_ (*B*_DFT_ = 117 GPa, *G*_DFT_ = 64 GPa)^19^ and has mechanical properties closer to that of the lithium antiperovskites (e.g., Li_3_OBr, *B*_DFT_ = 50.6 GPa, *G*_DFT_ = 37 GPa). Li_6_SiO_4_Cl_2_ is stiffer than related sulfide compounds (e.g., Li_6_PS_5_Br: *B*_DFT_ = 27 GPa, *G*_DFT_ = 14 GPa) and consequently easily meets the Monroe–Newman criterion for the prevention of dendrite growth.^47^ Future electrolytes based on these mixed oxide/halide compounds may thus be able to overcome some of the processing challenges and mechanical stability issues inherent in the use of pure oxide and sulfide ceramics.^48^

### 3.2 Synthesis, Thermal Behavior, and Structure Determination

**Figure 4:**
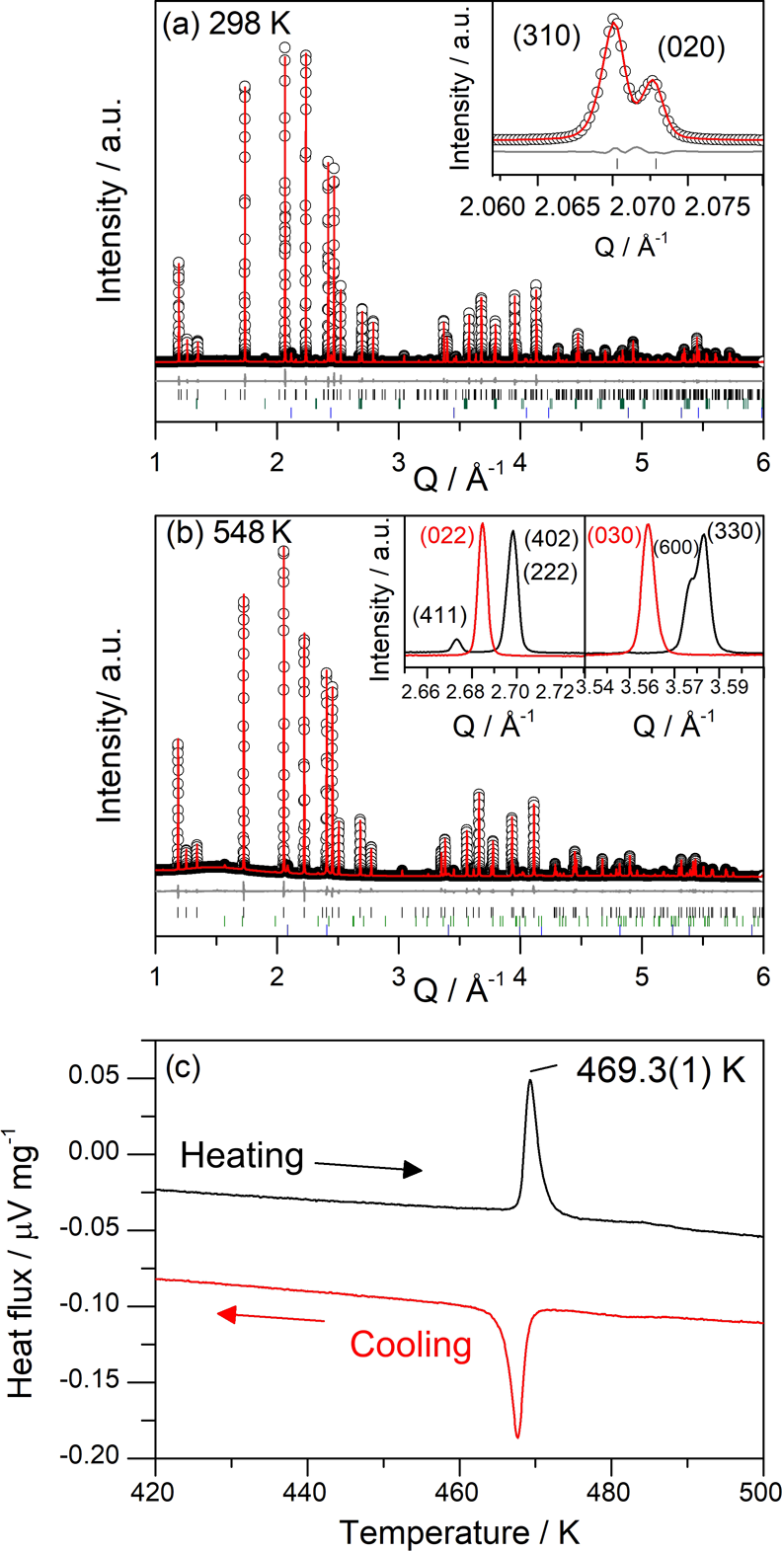
(a) RT-Li_6_SiO_4_Cl_2_: Rietveld refinement against SXRD of RT-Li_6_SiO_4_Cl_2_ (Diamond Light Source I11 beamline) with *I*_obs_ (black circles), *I*_calc_ (red line), *I*_obs_ – *I*_calc_ (gray line), and Bragg reflections (black tick marks for Li_6_SiO_4_Cl_2_, green tick marks for Li_2_SiO_3_, blue tick marks for LiCl); the inset highlights reflections consistent with the orthorhombic *Pna*2_1_ symmetry. (b) HT-Li_6_SiO_4_Cl_2_: Rietveld refinement against SXRD data of Li_6_SiO_4_Cl_2_ (black tick marks for Li_6_SiO_4_Cl_2_, green tick marks for LiAlO_2_, and blue tick marks for LiCl); the inset compares reflections from the RT-orthorhombic phase at 298 K (black line) with the HT-hexagonal phase at 548 K (red line). (c) Differential scanning calorimetry (DSC) data showing a reversible thermal event associated with phase transition between *Pna*2_1_ and *P*6_3_*mc* symmetries in Li_6_SiO_4_Cl_2_.

Compounds were synthesized as powders with compositions Li_6_SiO_4_Cl_2–*x*_Br*_x_* (*x* = 0, 0.5,1) to explore the computationally predicted targets. Synthesis of powders was attempted at varying reaction temperatures (50 K steps from 723 to 873 K) and reaction times (12, 24, 48 h), and starting materials and resulting powders were handled under an argon atmosphere and annealed in alumina crucibles in evacuated silica ampoules (Section 2.2). Synthesis at 823 K for 48 h yielded phase-pure powders for Li_6_SiO_4_Cl_2_. This new phase persists in the compositional range Li_6_SiO_4_Cl_2–*x*_Br*_x_* (0 ≥ *x* ≥ 1) and displays increased lattice parameters as a function of bromine content (Figure S1). Despite this clear indication of anion substitution, all compositions with *x* > 0 contained impurities; only Li_6_SiO_4_Cl_2_ was synthesized as a phase-pure white powder, and as such, all further discussion therefore concerns Li_6_SiO_4_Cl_2_. Single crystals of Li_6_SiO_4_Cl_2_ were synthesized by annealing at 883 K for 3 h before slow cooling (3 K h^–1^) to room temperature. High-resolution synchrotron XRD data (SXRD) were collected on the powder from 298 to 798 K in 25 K steps. At temperatures 473–498 K, convergence of some peaks and disappearance of other small peaks indicate a phase transition to a high-temperature, higher-symmetry phase (denoted HT phase) (Figure 4a,b). This transition is also observed in differential scanning calorimetry (DSC) data measured from Li_6_SiO_4_Cl_2_ powder, which shows endothermic and exothermic events on heating and cooling, respectively, associated with this reversible phase transition (Figure 4c). The exact transition temperature determined from DSC data is 469.3(1) K, consistent with the observations from SXRD, NMR, and AC impedance measurements (see below). Similarly to the RT phase, the experimental SXRD pattern of the HT phase could not be indexed to any known compounds in the Li–Si–O–Cl phase field. The structure of the RT phase was solved by single-crystal (SC) X-ray diffraction and the HT phase by the Rietveld refinement of a computed starting model against high-resolution SXRD data.

#### 3.2.1 Room-Temperature Phase: Structure Determination

The crystal structure was solved in the space group *Pna*2_1_ from a nonmerohedrally twinned crystal with lattice parameters: *a* = 10.5204(8) Å, *b* = 6.0756(4) Å, and *c* = 9.9530(7) Å. The assignments of Li, Si, O, and Cl were determined based on interatomic distances and relative displacement parameters. All atomic positions were refined with fixed fully occupied sites. Lithium atoms exhibited nonpositive definite anisotropic mean square displacements, so ISOR restraints were applied to atoms Li1–6 during the final refinement and also to atoms O2–4 as a result of distorted thermal ellipsoids. The final anisotropic atomic refinement converged to *R*_1_ = 0.0530, w*R*_2_ = 0.1189 for reflections with *I* ≥ 2σ (*I*) and *R*_1_ = 0.0750, w*R*_2_ = 0.1279 for all reflections. Crystallographic data and structural refinements for Li_6_SiO_4_Cl_2_ are summarized in Table S2. The asymmetric unit contains two distinct crystallographic Cl positions, four distinct O positions, one Si position (further confirmed by ^29^Si solid-state NMR, which displays a main signal at −67 ppm, Figure S2, corresponding to a SiO_4_^4–^ unit), and six Li positions. The final refined atomic positions, and isotropic and anisotropic displacement parameters of each atom are given in Tables S3 and S4, and the selected bond lengths and angles are given in Tables S5 and S6. Orthorhombic superlattice reflections characteristic of the *Pna*2_1_ (e.g., (411)) symmetry are clearly visible in the diffraction image, ruling out possible C-centered orthorhombic or hexagonal (as *a* ≈ √3*b*) supergroups.

The high-resolution room-temperature SXRD data further confirms this model showing subtle peak splitting consistent with the *Pna*2_1_ symmetry (Figure 4a, inset) evident due to higher *Q* resolution in the synchrotron data.

The observed systematic absences in the SXRD data were consistent with the *Pna*2_1_ space group and the lattice parameters refined to *a* = 10.543155(5) Å, *b* = 6.07657(3) Å, and *c* = 9.960255(5) Å from a Pawley fit. The model obtained from SC diffraction proved to be a good starting point for the Rietveld refinement with values of *R*_wp_ = 4.61 and χ^2^ = 2.81 after the initial refinement of site occupancies and displacement parameters. Cl and O site occupancies were refined, and Li site occupancies were fixed to achieve charge neutrality. Refinement of atomic positions improved *R*_wp_ from 4.61 to 4.27 and χ^2^ from 2.81 to 2.61 (Figure S3). Small impurity phases of LiCl (1.95%) and Li_2_SiO_3_ (2.35%) were observed (Figure S4a). The maximum entropy method (MEM) analysis did not show any additional electron density, indicating that all lithium sites are accounted for in the structural model described above (Figure S6). The final Rietveld fit is shown in Figure 4a, and refined structural parameters are presented in Tables S7 and S8. The refined composition is Li_5.957(2)_Si_1.00_O_3.986(2)_Cl_1.985(1)_, and the RT phase will therefore be denoted RT-Li_6_SiO_4_Cl_2_ hereafter for simplicity.

#### 3.2.2 Room-Temperature Phase: Structure Description

As described in Section 3.1, the orthorhombic structure arises from the rotation of lithium atoms off the mirror plane in *P*6_3_*mc* through activation of Γ_5_ and M_2_ modes, stabilizing the *Pna*2_1_ symmetry. This leads to a change in anionic and cationic coordination environments compared to the hexagonal defect antiperovskite parent.

**Figure 5:**
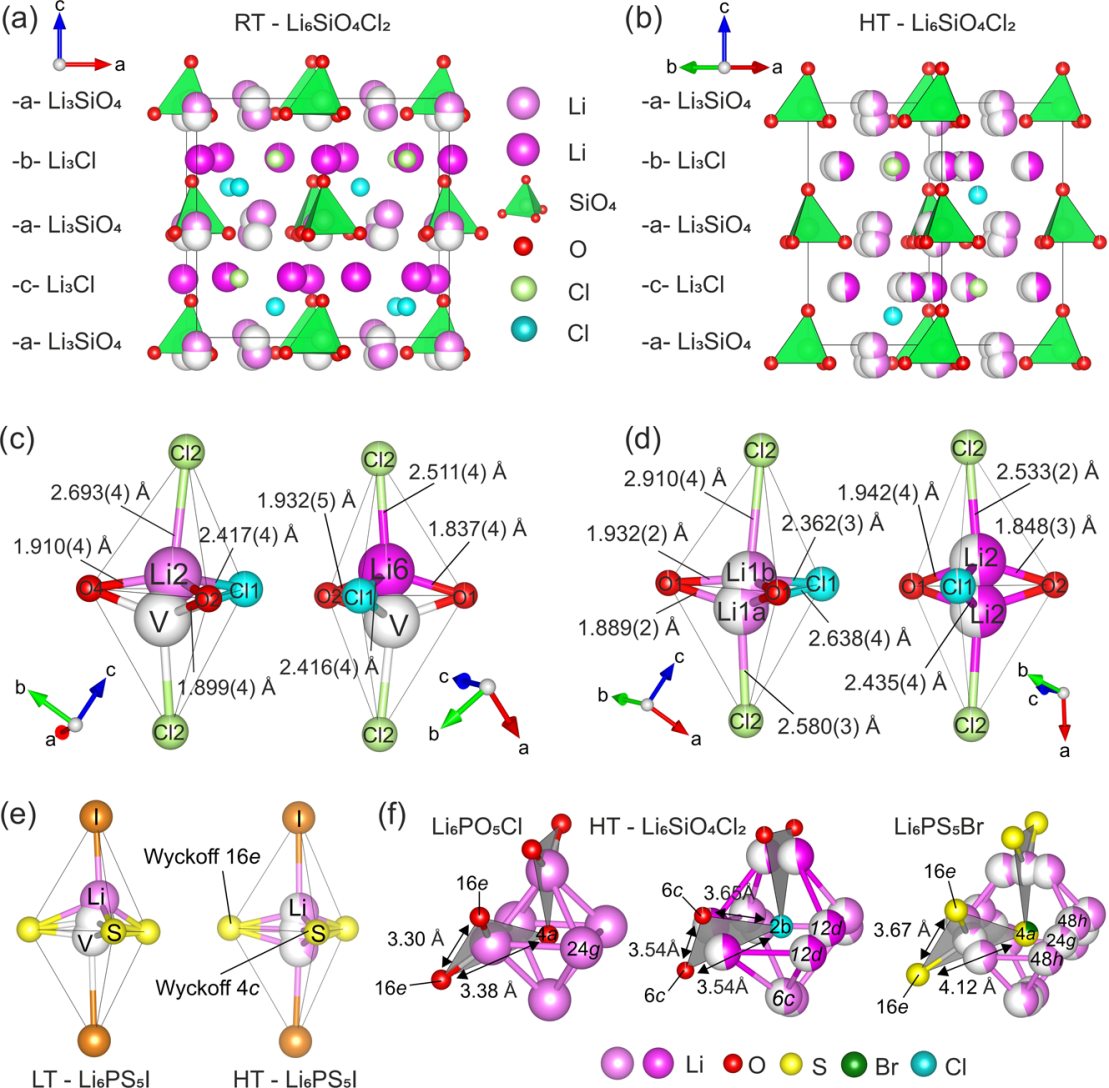
(a) RT-Li_6_SiO_4_Cl_2_ unit cell showing alternating Li_3_Cl and Li_3_SiO_4_ layers. Comparing with the ABX_3_ perovskite, SiO_4_ (green tetrahedra) and Cl2 (light green) occupy the A-sites, Cl1 (blue) ions fill 50% of the B-sites with the remaining 50% vacant, and lithium atoms occupy the X-sites (Li1, Li2, Li3 atoms in light pink, Li4, Li5, Li6 atoms in dark pink). Vacant tetrahedral lithium sites in the Li_3_SiO_4_ layer are shown in white to facilitate comparison with the HT structure. (b) HT-Li_6_SiO_4_Cl_2_ unit cell showing the same alternating Li_3_Cl and Li_3_SiO_4_ layers. Lithium atoms are disordered over partially occupied sites with respect to the RT structure (Li1a and Li1b atoms in light pink, Li2 atoms in dark pink). (c) RT-Li_6_SiO_4_Cl_2_ lithium tetrahedra showing displacement of lithium (4a Wyckoff site) toward coordinating oxygen. The vacant tetrahedral sites (V), drawn in white, are generated by activation of occupational modes when lowering the symmetry of HT-Li_6_SiO_4_Cl_2_ to *Pna*2_1_ through ISODISTORT (Figure S10). (d) HT-Li_6_SiO_4_Cl_2_ Li1a, Li1b (6*c* Wyckoff site), and Li2 (12*d* Wyckoff site) coordination environment, showing partial occupancy of tetrahedral sites that are both fully occupied and vacant at RT. (e) This order–disorder behavior of lithium sites is analogous to the lithium distribution in Li_6_PS_5_I argyrodite;^21^ at low temperatures (LTs), lithium atoms are ordered occupying one of the tetrahedral positions, whereas at high-temperature (HT), lithium ions are disordered with partial occupancy of tetrahedral and trigonal positions. Distinct sulfide anion positions are labeled in the HT structure. (f) Octahedral Li-ion cages surrounding B-site anions in Li_6_PO_5_Cl (cubic), HT-Li_6_SiO_4_Cl_2_ (hexagonal), and Li_6_PS_5_Br (cubic). Trigonal anion windows (consisting of three oxide anions in Li_6_PO_5_Cl, two oxide anions and one chloride anion in Li_6_SiO_4_Cl_2_, and two sulfide and one mixed sulfide/bromide site in Li_6_PS_5_Br) are shown, highlighting their anion–anion distances that determine the window area. Two distinct trigonal windows are present in HT-Li_6_SiO_4_Cl_2_, with oxide ions both either occupying Wyckoff position 6*c* (edge length: 3.54 Å) or occupying Wyckoff positions 2*a* and 6*c* (edge length: 3.23 Å). Lithium ions move through these windows in argyrodite solid electrolytes.

The experimentally determined RT structure is shown in Figure 5a, highlighting the atomic packing within the structure defined by alternating Li_3_SiO_4_ and Li_3_Cl layers. Comparison of the experimentally determined *Pna*2_1_ structure to the computed structure shows almost identical atomic positions (Figure S7a). The chloride ions that occupy 50% of the antiperovskite B-site positions (Cl1) are found in an octahedral environment with bond lengths ranging from 2.415(3) to 2.621(3) Å to neighboring Li. Compared to the idealized octahedral environment expected for B-site anions in antiperovskites, the octahedra are distorted slightly as a result of the nonrigid rotation of Li ions and the displacement of chlorine toward neighboring vacant B-sites. The chloride ions that occupy 50% of the A-site (Cl2), typically forming cuboctahedral (12-fold) coordination environments in hexagonal perovskites such as 4H-BaMnO_3_, occupy a distorted octahedral environment in the orthorhombic polymorph with bond lengths from 2.446(3) to 2.798(7) Å (Figure S8). All lithium atoms are found in tetrahedral LiCl_2_O_2_ coordination environments. Due to the difference in ionic radii (*r*_Cl_ = 1.81 Å, *r*_O_ = 1.40 Å),^45^ lithium atoms are displaced toward the oxygen atoms (Figure 5c). Li–O bond lengths vary from 1.829(3) to 1.931(3) Å, comparable to distances reported for the cubic argyrodite Li_6_PO_5_Cl (1.93 Å)^9^ and shorter than Li–O bonds in Li_4_SiO_4_ (1.84–2.51 Å).^49^ Li–Cl bond lengths range from 2.415(3) to 2.798(3) Å. These values are larger than expected for typical LiCl_4_ bond lengths, e.g., 2.38 Å in Li_2_MgCl_4_.^50^ The difference in bond lengths compared to homoleptic LiCl_4_ and LiO_4_ tetrahedral environments is expected in heteroleptic LiCl_2_O_2_ and optimizes the bond valence sum for Li^+^ (BVS: 0.939(8)–1.12(1)). The Li–Cl distances in Li_6_SiO_4_Cl_2_ are shorter than in the cubic oxide argyrodite Li_6_PO_5_Cl (2.910(4) Å), in which Li atoms have a trigonal bipyramidal coordination (LiO_3_Cl_2_) with Cl occupying the axial positions.^9^

**Figure 6:**
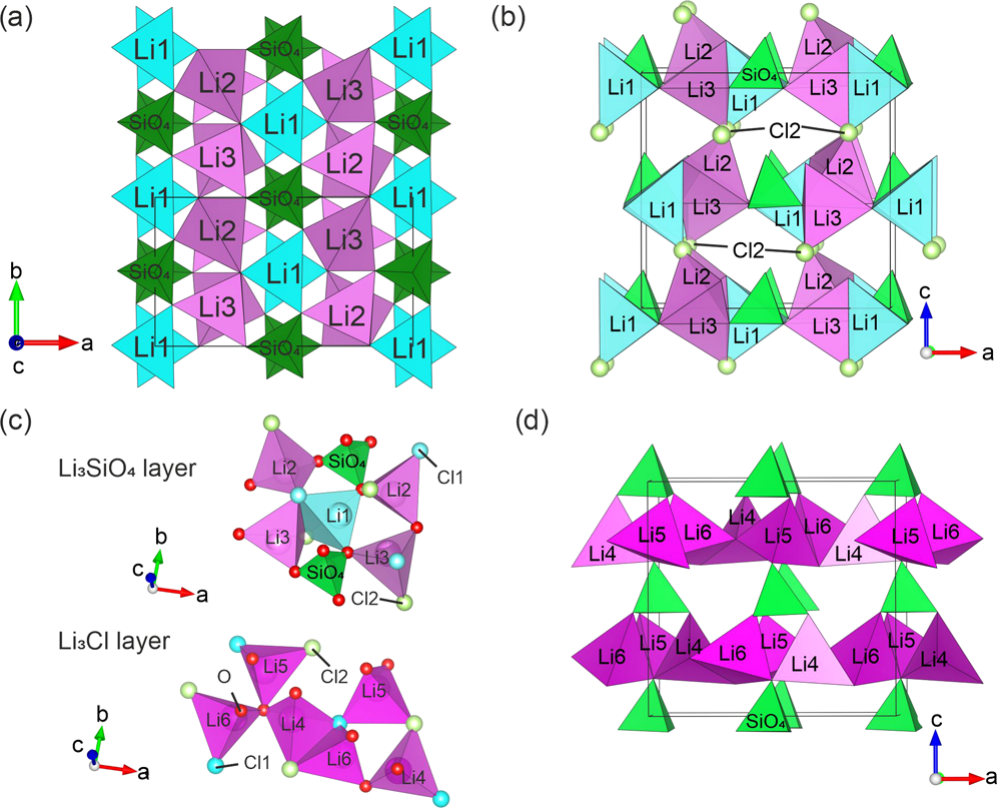
(a) Li_3_SiO_4_ intralayer environments and Li2–Li3 (pink) and Li1–SiO_4_ (blue and green, respectively) chains of corner- and edge-sharing tetrahedra. (b) Connection of Li_3_SiO_4_ layers along the stacking axis (c) through corner-sharing of Cl2 atoms (light green). (c) Li_3_SiO_4_ (top) and Li_3_Cl (bottom) layers consisting of corner- and edge-sharing tetrahedra (Li1: light blue, Li2, Li3: light pink, Li4–Li6: dark pink, Si: green, Cl1 (B-sites): blue, Cl2 (A-sites): light green, O: red), all shared edges are Cl–Cl. (d) Li_3_Cl layers connected via SiO_4_ units viewed along the *b*-direction showing stacking along c; SiO_4_ tetrahedra are shown to guide the eye.

In the RT structure, LiCl_2_O_2_ tetrahedra are connected through the corner-sharing of Cl and O vertices and the sharing of Cl–Cl edges; they are referred to using the central lithium atom, i.e., Li(1)O_2_Cl_2_ as a Li1 tetrahedron. Edge-sharing between Li1–Li3 and Li4–Li6 tetrahedra is facilitated by the lithium-ion displacements away from the shared Cl–Cl edges (Figure 6a,c), minimizing electrostatic repulsions.

The Li_3_SiO_4_ layer can be described (Figure 6a) as alternating corner-sharing Li2–Li3 and Li1–SiO_4_ chains running along b, connected in the *a*-direction by Li1–Li2, SiO_4_–Li2, SiO_4_–Li3 corner-sharing, and Li1–Li3 edge-sharing (Figure 6b,c). Cl2 atoms located in the Li_3_Cl layers connect Li_3_SiO_4_ layers along the stacking *c*-direction (Figure 6b). The Li_3_Cl layer is made up of Li4, Li5, and Li6 tetrahedra connected through Li4–Li5, Li4–Li6, Li5–Li6 corner-sharing, and Li4–Li6 edge-sharing (Figure 6c,d). The Li_3_Cl layer is connected to the Li_3_SiO_4_ layer through corner-sharing with Li1, Li2, Li3, and SiO_4_ tetrahedra, forming the three-dimensional (3D)-network of corner- and edge-shared tetrahedra.

#### 3.2.3 High-Temperature Phase: Structure Determination

Indexing of the HT phase (above 523 K) with TOPAS resulted in a hexagonal unit cell with lattice parameters *a* = 6.11 Å and *c* = 10.02 Å. Systematic absences in the powder pattern were consistent with the *P*31*c* and *P*6_3_*mc* space groups, agreeing with the computationally stable hexagonal structures (Section 3.1 and Figure 3c). Very similar Pawley fits were obtained using these symmetries, and lattice parameters were refined to *a* = 6.110805(12) Å and *c* = 10.02068(3) Å for *P*6_3_*mc* (Figure S4b). The higher-symmetry computed *P*6_3_*mc* structure gave a reasonable starting point for the Rietveld refinement. The unit cell contains two distinct crystallographic Cl positions (Wyckoff position 2*b*), two O positions (Wyckoff positions 6*c* and 2*a*), one Si position (Wyckoff position 2*a*), and three Li positions (Wyckoff positions 6*c* and 12*d*). Occupancies of Cl and O sites were refined, and the occupancies of Li sites were fixed to ensure charge neutrality. In the computational model, Li2 is located on the mirror plane in special position 6*c* (Figure S7b). Inspection of the Fourier density map (FDM) reveals extra electron density around this position and indicates displacement of Li2 away from the mirror plane onto the general position 12*d* (Figure S9a). Replacing the 6*c* site with the 12*d* position improved *R*_wp_ from 3.61 to 2.98%. The FDM also revealed electron density around the Li1 position along the *c*-direction (Figure S9b). Splitting the Li1 site into two distinct Li1a and Li1b sites along c further improved *R*_wp_ to 2.75%. The computed *P*31*c* model (Figure 3c) is similar, only allowing two distinct 6*c* crystallographic sites for Li2 instead of one 12*d* site. A fit of the diffraction data to this model, with independent refinement of both Li2a and Li2b 6*c* occupancies, did not result in an improved fit. The *P*6_3_*mc* model was therefore used for final refinement. MEM analysis also supports the displacement of Li1 and Li2 positions (Figure S6). The final refinement is shown in Figure 4b, and the refined structural parameters are provided in Table S7. The refined composition is Li_5.82(4)_Si_1.00_O_3.934(6)_Cl_1.956(4)_, and the HT phase will therefore be denoted HT-Li_6_SiO_4_Cl_2_ hereafter for simplicity.

#### 3.2.4 High-Temperature Phase: Structure Description

The HT *P*6_3_*mc* structure can be described as a 4H-hexagonal antiperovskite of general formula ABX_3_, with structural similarities to 4H-BaMnO_3_ (Figure 2b) in which half of the B-sites are vacant. This is consistent with cation-deficient hexagonal perovskites with the formula A_2_BX_6_, such as Rb_2_MnF_6_ with the *P*6_3_*mc* symmetry.^51^ The HT structure of Li_6_SiO_4_Cl_2_, consisting of alternating hexagonally stacked Li_3_Cl and Li_3_SiO_4_ layers, is shown in Figure 5b. The (poly)anion network in the HT structure remains unchanged through the phase transition from the RT structure, with the observed symmetry change originating from displacements in Li positions. The lithium environments remain tetrahedral (LiO_2_Cl_2_) with lithium atoms displaced toward the oxygens due to its smaller ionic radius compared to chlorine. Li–Cl bond lengths vary from 2.366(3) to 2.908(8) Å, and Li–O bond lengths range from 1.847(4) to 1.942(4) Å (Figure 5d).

As shown in Figure 5c,d, the ordered lithium atoms of RT-Li_6_SiO_4_Cl_2_ are disordered in the higher-symmetry HT structure. Similar to the results from normal mode calculations described in Section 3.1, this order–disorder transition occurs via activation of occupational M_2_ and Γ_5_ modes, with a small contribution from displacive Γ_5_ modes that predominantly involve displacement of the Li1 atoms within the close-packed Li_3_SiO_4_ layer (Figure S10). This disorder of the *A* cation positions is well known in *A*_(12–n*–*y*)/*m*_^*m*+^*L*_6–*y*_^*n*+^*X*_6–*y*_^2–^*Y*_*y*_^–^ argyrodites^52,53^ and provides a direct comparison of Li disorder between oxide and sulfide argyrodite materials. The delocalization of Li positions in HT-Li_6_SiO_4_Cl_2_ resembles that observed in high-temperature *F*4̅3*m* cubic argyrodites Li_6_PS_5_X (X = Cl, Br, I), in which Li partially occupies both tetrahedral and trigonal planar coordination environments (48*h* and 24*g* Wyckoff sites, respectively; Figure S9c) and is one of the reasons for reduced energy barriers for bulk Li-ion transport in these materials.^8^

The order–disorder transition of Li_6_SiO_4_Cl_2_ (Figure 5c,d) is similar to the behavior of many argyrodites; Cu_8_GeSe_6_ displays an order–disorder transition between *P*6_3_*cm* and *P*6_3_*mc* structures, where Cu ions are disordered in the latter similarly to Li on the 6*c* and 12*d* Wyckoff positions in Li_6_SiO_4_Cl_2_,^39,54^ and analogous behavior is observed in silver-containing argyrodites.^1^ Specifically, cubic Li_6_PS_5_I has ordered lithium positions at low temperature (<180 K) and a disordered structure at 298 K in which lithium is delocalized across the 24*g* and 48*h* positions (Figure 5e),^21^ resulting in comparable local lithium coordination environments with Li_6_SiO_4_Cl_2_ (Figure 5c,d) that are consistent with dynamical disorder. This is distinct from the cubic oxide argyrodite, Li_6_PO_5_Br, in which lithium remains localized on the trigonal planar 24*g* Wyckoff position in the *F*4̅3*m* structure over a wide (173–873 K) temperature range (Figure 1e).^48^ Stabilization of this lithium disorder, which enables access to higher-energy sites in Li_6_SiO_4_Cl_2_, would increase lithium-ion migration compared against the room-temperature structure.

### 3.3 Transport Properties

**Figure 7:**
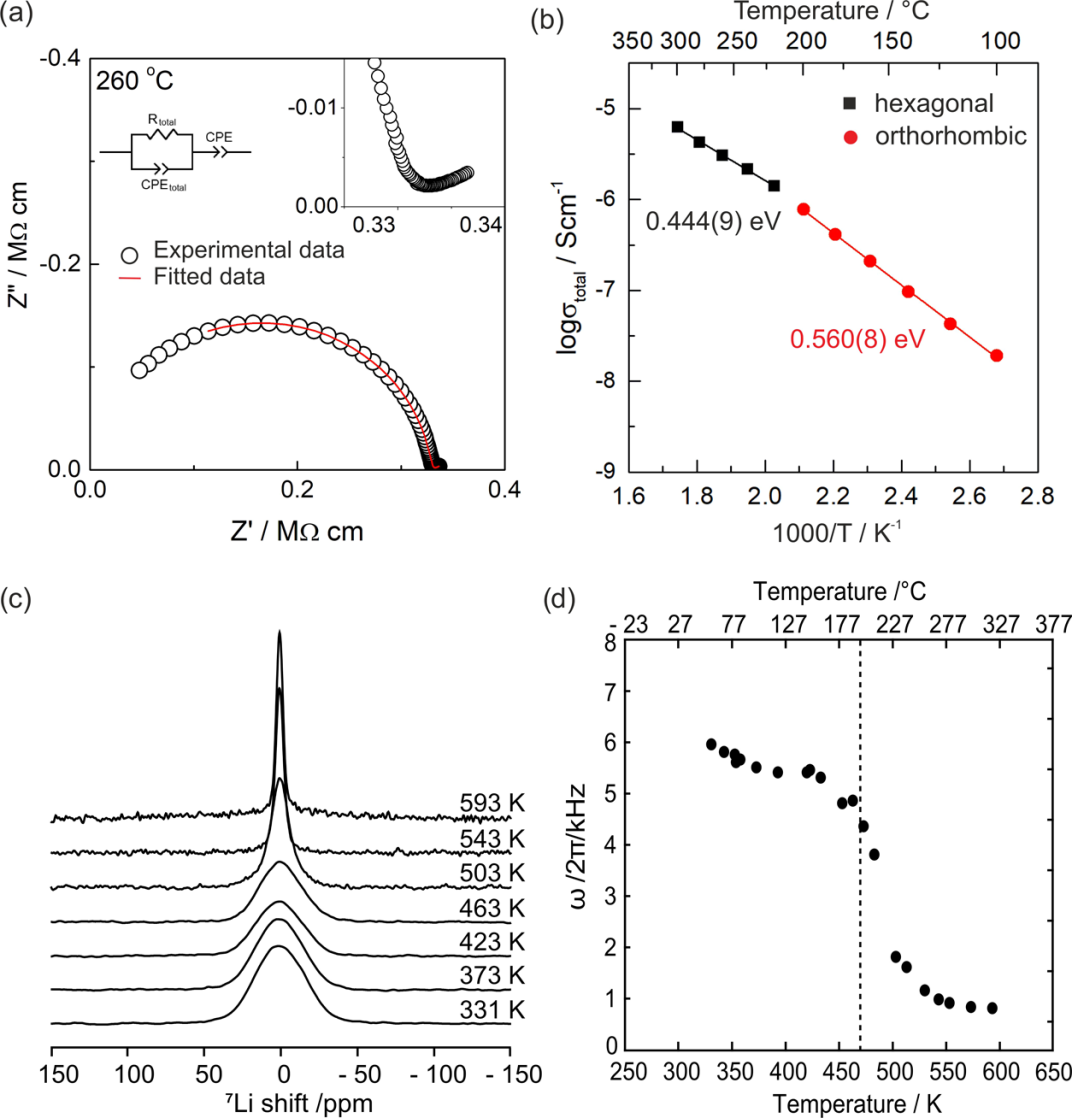
(a) Impedance complex plane plots Z* of Li_6_SiO_4_Cl_2_ at 533 K; the inset shows equivalent circuit used to model the data. The inset shows the low-frequency inclined spike attributed to the capacitance of the blocking electrode. (b) Arrhenius plots of the total conductivity; activation energies derived from the data are shown. A change in Arrhenius behavior and activation energy can be seen at ∼470 K corresponding to the phase change observed in VT-XRD and DSC measurements. (c) ^7^Li NMR spectra under static conditions as a function of temperature. (d) Motional narrowing of the line width (full width at half-maximum) of the central ^7^Li NMR transition. The temperature at which the phase change is detected via DSC measurements is also noted (dashed line).

The ionic conductivity of Li_6_SiO_4_Cl_2_ was investigated through AC impedance spectroscopy on a sintered white pellet of ∼84% theoretical density (pellet cold-pressed under 125 MPa and sintered in an evacuated silica ampoule for 12 h at 848 K). A typical set of data measured at 533 K in an inert atmosphere are shown in Figure 7a. The impedance complex plane plots, Z*, consist of a high-frequency arc with the presence of a small low-frequency inclined spike. The large single arc is attributed to the sample bulk, as shown by overlapping peaks in the combined Z″/M″ spectra (Figure S12a). The associated capacitance of the high-frequency arc, 0.6 pF cm^–1^, corresponding to a permittivity of ∼6.8, is also consistent with the grain response (Figure S12b). The low-frequency spike is attributed to the double-layer capacitance at the blocking sample–electrode interface, where Li ions cannot pass. To a first approximation, the high-frequency arc could be modeled with an equivalent circuit consisting of a resistor in parallel with a constant phase element (CPE). Impedance data were fitted to the equivalent circuit using ZView software (Figure 7a). In general, the material is homogeneous and shows a total conductivity of 6.2 × 10^–6^ S cm^–1^ at 575 K and ∼10^–10^ S cm^–1^ at room temperature. DC polarization measurements show a low electronic contribution of >0.5% at 300 °C to the overall conductivity (eq 1 and Figure S13). From the low-frequency intercept of the impedance arc on the *Z*′ axis, the values of the total resistance were obtained and are shown in an Arrhenius format in Figure 7b. A change in slope can be observed above ∼498 K, agreeing with the phase transition observed via VT-XRD and DSC experiments. The orthorhombic phase (red circles) and the hexagonal phase (black squares) have activation energies of 0.560(8) and 0.444(9) eV, respectively.

The local ionic mobility of lithium was investigated through ^7^Li solid-state NMR. The temperature dependence of the static ^7^Li NMR spectra of Li_6_SiO_4_Cl_2_ over the temperature range of 331–593 K is shown in Figure 7c. At temperatures where ion mobility is minimal, i.e., the rigid lattice regime, the 1/2 ↔ −1/2 central transition is broadened by the ^7^Li–^7^Li homonuclear dipolar coupling. For Li_6_SiO_4_Cl_2_, this region is between 350 and 450 K, where the line width of the central transition is ∼5.5 kHz. As the temperature is increased above 450 K, the line width decreases drastically over a small temperature range (Figure 7d) due to the increasing motion of the Li spins continuously averaging the dipolar interactions and clearly evidences an increase in ionic mobility facilitated by the phase transition from the ordered RT-orthorhombic to the higher-symmetry HT-hexagonal phase.

Through calculation of the strength of ^7^Li–^7^Li homonuclear dipolar coupling of both the room-temperature and high-temperature phases of Li_6_SiO_4_Cl_2_, further insight into the local ion dynamics of the two phases can be determined. By extracting the shortest Li–Li distance from the SXRD data (Li1–Li5, 2.697(6) Å), the absolute value of the dipolar coupling constant can be calculated (eq 2) as ∼5.8 kHz in RT-Li_6_SiO_4_Cl_2_ in full agreement with the experimental line width observed, while ∼8.2 kHz is obtained from Li1a and Li2 (2.410(5) Å) for HT-Li_6_SiO_4_Cl_2_. Due to the increase in dipolar coupling after the phase transition, it would be expected that ^7^Li spectra demonstrate increased line widths at higher T in the absence of an increase in Li-ion mobility. However, the line narrowing observed (Figure 7d) is a clear indication that the phase transition from the RT-orthorhombic phase to the higher-symmetry HT-hexagonal phase facilitates an increase in Li-ion mobility, as also observed in AC impedance measurements (Figure 7b). The delocalization of lithium in HT-Li_6_SiO_4_Cl_2_ across the 6*c* and 12*d* sites (Figure 5c,d) results in a lowering of the activation energy from RT-Li_6_SiO_4_Cl_2_ where the lithium positions are fully ordered. The activation energies obtained from Li_6_SiO_4_Cl_2_ are lower than those extracted from Li_6_PO_5_Cl across the measured temperature range, despite the latter adopting the higher-symmetry cubic *F*4̅3*m* structure.^9^

The distinction between the oxide materials Li_6_SiO_4_Cl_2_ and Li_6_PO_5_Cl is likely the increased size of the trigonal anion window (Figure 5f) and the associated dynamical Li disorder (absent in cubic Li_6_PO_5_Cl, where Li is localized on the 24*g* position) in hexagonal Li_6_SiO_4_Cl_2_ (partial occupancy of 6*c* and 12*d* positions by Li), leading to lower activation energies for ion mobility. Local jumps of Li^+^ between these tetrahedral sites separated by the trigonal anion windows are the most favorable for ionic diffusion in argyrodites.^8^ Occupational disorder via chalcogenide–halide mixing on the anion position that forms part of the trigonal window that mobile cations traverse (Figure 5f) is an important route to enhancing Li-ion mobility.^12,54^ Unlike Li_6_PO_5_Cl, where this window is formed from three oxide anions, the LiCl_2_O_2_ environments of Li_6_SiO_4_Cl_2_ yield a window described by one chloride and two oxide anions. The larger Cl (*r*_Cl_ = 1.81 Å, *r*_O_ = 1.40 Å)^45^ increases the size of this window, further lowering the activation energy of ion transport compared to Li_6_PO_5_Cl.

## 4 Conclusions

The connection between argyrodite and antiperovskite structures is established, thus identifying close-packed layers that can be assembled through the diversity of combined hexagonal and cubic stacking operations known to give the perovskite material family its breadth of scientific and technological importance. We exemplify these mixed stackings in the hexagonal argyrodite material family through the prediction and then isolation of the nontoxic and earth-abundant Li_6_SiO_4_Cl_2_. Here, the resulting layer stacking gives access to a disordered Li distribution, which is observed in fast lithium-ion conductors, a distribution not accessible to its nearest known cubic compositional counterpart, Li_6_PO_5_Cl. Exploration of the many accessible mixed layer sequences and associated site and chemical orderings afforded within this family opens new routes to the tuning and creation of ion transport pathways through variation of both composition and structure, as well as broader functional outcomes based on the importance of the cubic argyrodite materials.

## Supporting Information Available

Computed decomposition energies of the compounds Li_6_SiO_4_XX′ (X = F, Cl, Br, I) for different space groups, PXRD patterns for Li_6_SiO_4_Cl_2–*x*_Br*_x_*, Pawley fits (RT and HT phases), comparison of Rietveld refinement before and after refinement of atomic positions as well as with and without Li atoms (RT phase), MEM analysis (RT and HT phases), comparison of computed and experimental structures (RT and HT phases), Fourier difference map (HT phase), structural information of Li_6_SiO_4_Cl_2_ determined by single-crystal diffraction and powder diffraction refinement (refinement details, atomic positions, bond distances, and angles), ^29^Si NMR spectrum and impedance results are available (PDF)
Crystal structure of SC-Li_6_SiO_4_Cl_2_ is deposited with ICSD 2032155 (CIF)
Crystallographic data (RT-Li_6_SiO_4_Cl_2_-powder-responses) (CIF)
Crystallographic data (HT-Li_6_SiO_4_Cl_2_) (CIF)

## Supplementary Material

cm1c00157_si_001.pdf

cm1c00157_si_002.cif

cm1c00157_si_003.cif

cm1c00157_si_004.cif
