# Supplementary material for: Li6SiO4Cl2: A Hexagonal Argyrodite Based on Antiperovskite Layer Stacking
Source: Chem Mater. 2021 Mar 2;33(6):2206–17. doi: 10.1021/acs.chemmater.1c00157 (PMC8029579; doi:10.1021/acs.chemmater.1c00157)
Supplement: Supplementary file 1 — cm1c00157_si_001.pdf [file cm1c00157_si_001.pdf]

Supplementary Information

$Li_6SiO_4Cl_2$: A Hexagonal Argyrodite based on anti-perovskite layer stacking

Alexandra Morscher,[a] Matthew S. Dyer,[a] Benjamin B. Duff,[a,b] Guopeng Han,[a] Jacinthe Gamon,[a] Luke M. Daniels,[a] Yun Dang,[a] T. Wesley Surta,[a] Craig M. Robertson,[a] Frédéric Blanc,[a,b] John B. Claridge[a] and Matthew J. Rosseinsky*[a]

[a] Department of Chemistry, University of Liverpool, Crown Street, L69 7ZD Liverpool, UK.

[b] Stephenson Institute for Renewable Energy, University of Liverpool, Peach Street L69 7ZF Liverpool, UK.

*Corresponding Author: m.j.rosseinsky@liverpool.ac.uk

Content

1.	Computational study	Table S1	Page 2
2.	Structural study	Figure S1 – S11<br>Table S2 – S9	Pages 3-16
3.	Ionic conductivity study	Figure S12	Page 17

1. Computational study

Table S1: Computed decomposition energies of the compounds Li_6SiO_4XX' in the $P6_3mc$ hexagonal argyrodite structure and its four lower symmetry structural derivatives: $Pna2_1$, $P31c$, $Pca2_1$ and $P6_3mc$. Where there are two distinct halides present, the smallest is placed in the B-site of the inverse hexagonal perovskite, and the larger in the Li_3X layer representing the A-site. Energies are computed relative to a stochiometric combination of lithium orthosilicate (Li_4SiO_4) and the relevant binary lithium halides (LiX and LiX′). Full convex hull calculations for the Li-Si-O-Cl-Br and Li-Si-O-Cl-I phase fields confirm that this is the most stable combination of previously reported compounds at this composition in those phase fields. Negative energies represent compositions which are more stable than Li_4SiO_4+LiX+LiX′, and the energy of the most stable structure for each composition is highlighted in bold type.

Compound	$P6_3mc$ (meV/atom)	$Pna2_1$ (meV/atom)	$P31c$ (meV/atom)	$Pca2_1$ (meV/atom)	$P6_3$ (meV/atom)
$Li_6SiO_4F_2$	56	46	66	**25**	56
$Li_6SiO_4Cl_2$	3	**-15**	-12	-10	-10
$Li_6SiO_4Br_2$	8	**-6**	-4	-3	-3
$Li_6SiO_4I_2$	55	48	**48**	48	48
Li_6SiO_4FCl	27	**22**	23	25	25
Li_6SiO_4ClBr	-8	**-16**	-14	-12	-13
Li_6SiO_4BrI	12	11	**10**	11	67
Li_6SiO_4FBr	18	**18**	18	18	18
Li_6SiO_4FI	31	**31**	31	31	31
Li_6SiO_4ClI	-2	-2	**-2**	-2	-2

2. Structural study

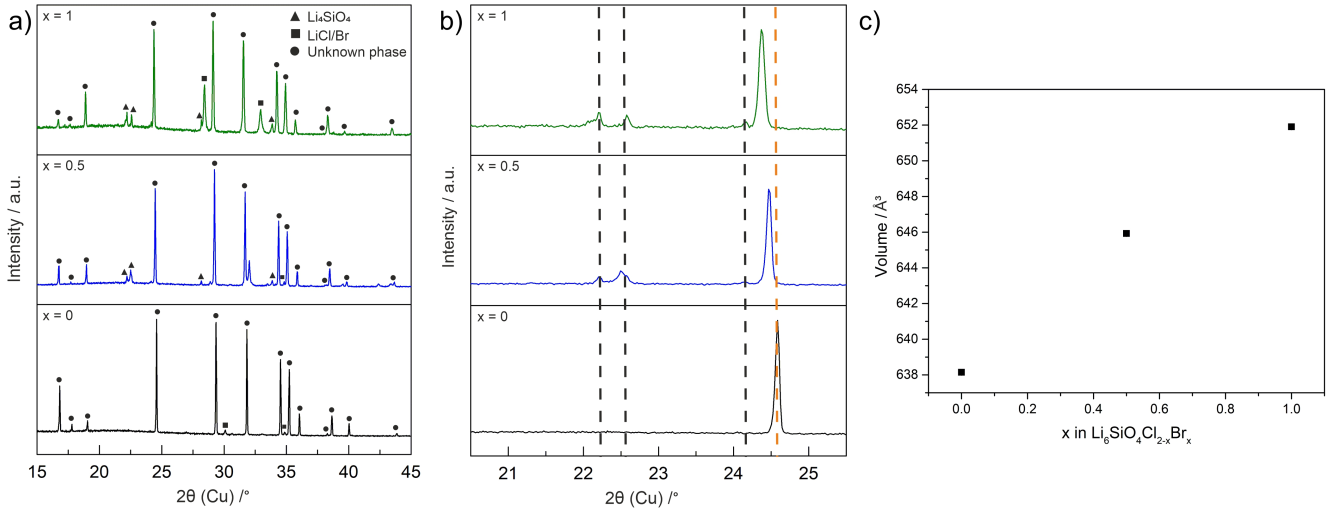

Figure S1: a) Laboratory PXRD patterns for $Li_6SiO_4Cl_{2-x}Br_x$ (x = 0, 0.5, 1) and b) PXRD patterns highlighting 2θ shift through incorporation of bromine (black lines correspond to Li_4SiO_4, orange lines to the new unindexed phase). c) Variation in unit cell volume as a function of bromine content x in $Li_6SiO_4Cl_{2-x}Br_x$. Error bars are smaller than the data points.

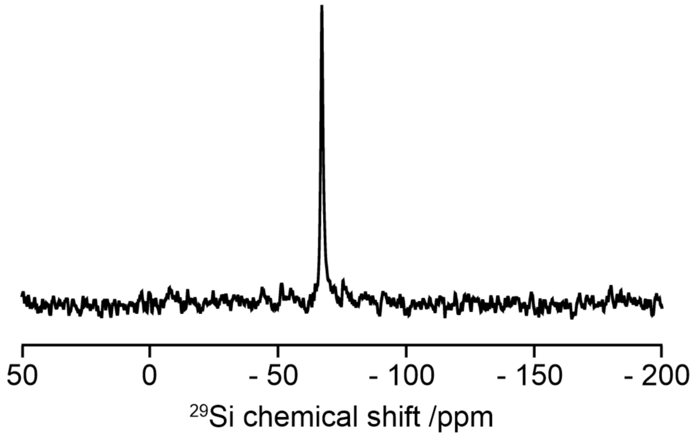

Figure S2: ^{29}Si MAS NMR spectrum of $Li_6SiO_4Cl_2$ at MAS rate v_r = 10 kHz

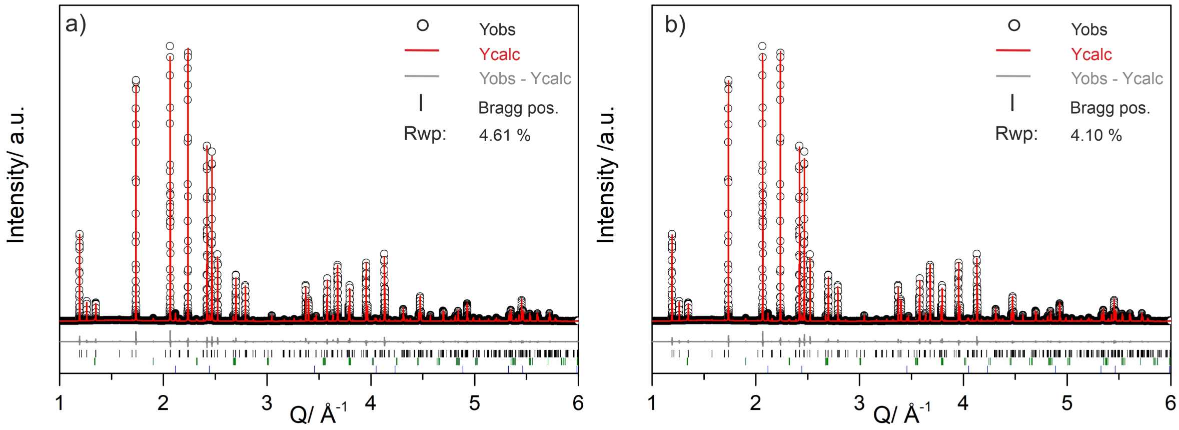

Figure S3: a) RT – $Li_6SiO_4Cl_2$ initial fit of SC-model without refinement of lattice sites with Y_{obs} (black circles), Y_{calc} (red line), Y_{obs} - Y_{calc} (grey line) and Bragg reflections (black tick marks for $Li_6SiO_4Cl_2$, green tick marks for Li_2SiO_3 and blue tick marks for LiCl) b) RT – $Li_6SiO_4Cl_2$ final Rietveld refinement after refinement of lattice sites.

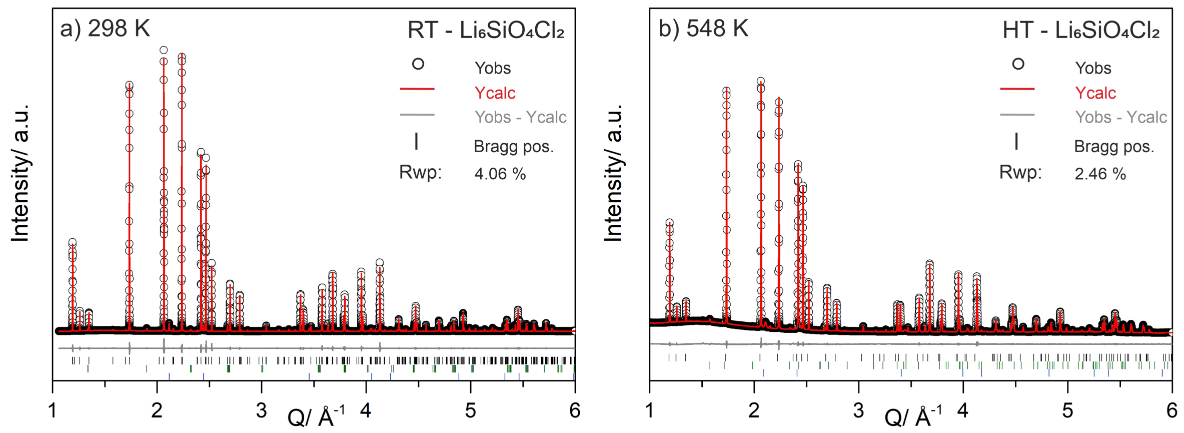

Figure S4: a) RT – $Li_6SiO_4Cl_2$ Pawley fit with *Pna*2_1 symmetry (black tick marks for $Li_6SiO_4Cl_2$, green tick marks for Li_2SiO_3, blue tick marks for LiCl) Y_{obs} (black circles), Y_{calc} (red line), Y_{obs} - Y_{calc} (grey line) and Bragg reflections b) HT – $Li_6SiO_4Cl_2$ Pawley fit to SXRD data with *P*6_3*mc* symmetry (black tick marks for $Li_6SiO_4Cl_2$, green tick marks for $LiAlO_2$, blue tick marks for LiCl).

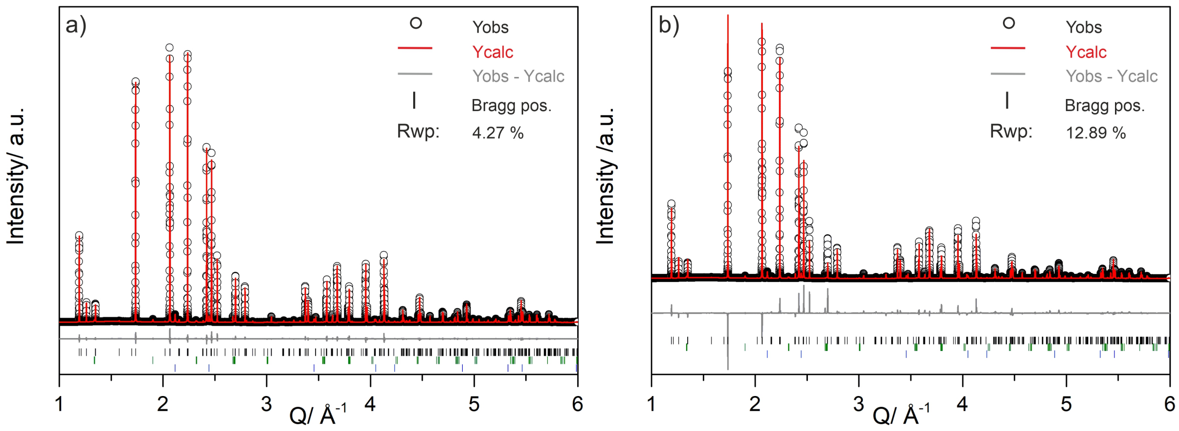

Figure S5: a) RT – $Li_6SiO_4Cl_2$: Final Rietveld refinement with Li positions included. Y_{obs} (black circles), Y_{calc} (red line), Y_{obs} - Y_{calc} (grey line) and Bragg reflections (black tick marks for $Li_6SiO_4Cl_2$, green tick marks for Li_2SiO_3 and blue tick marks for LiCl) b) RT – $Li_6SiO_4Cl_2$: Final Rietveld refinement with Li positions removed from model.

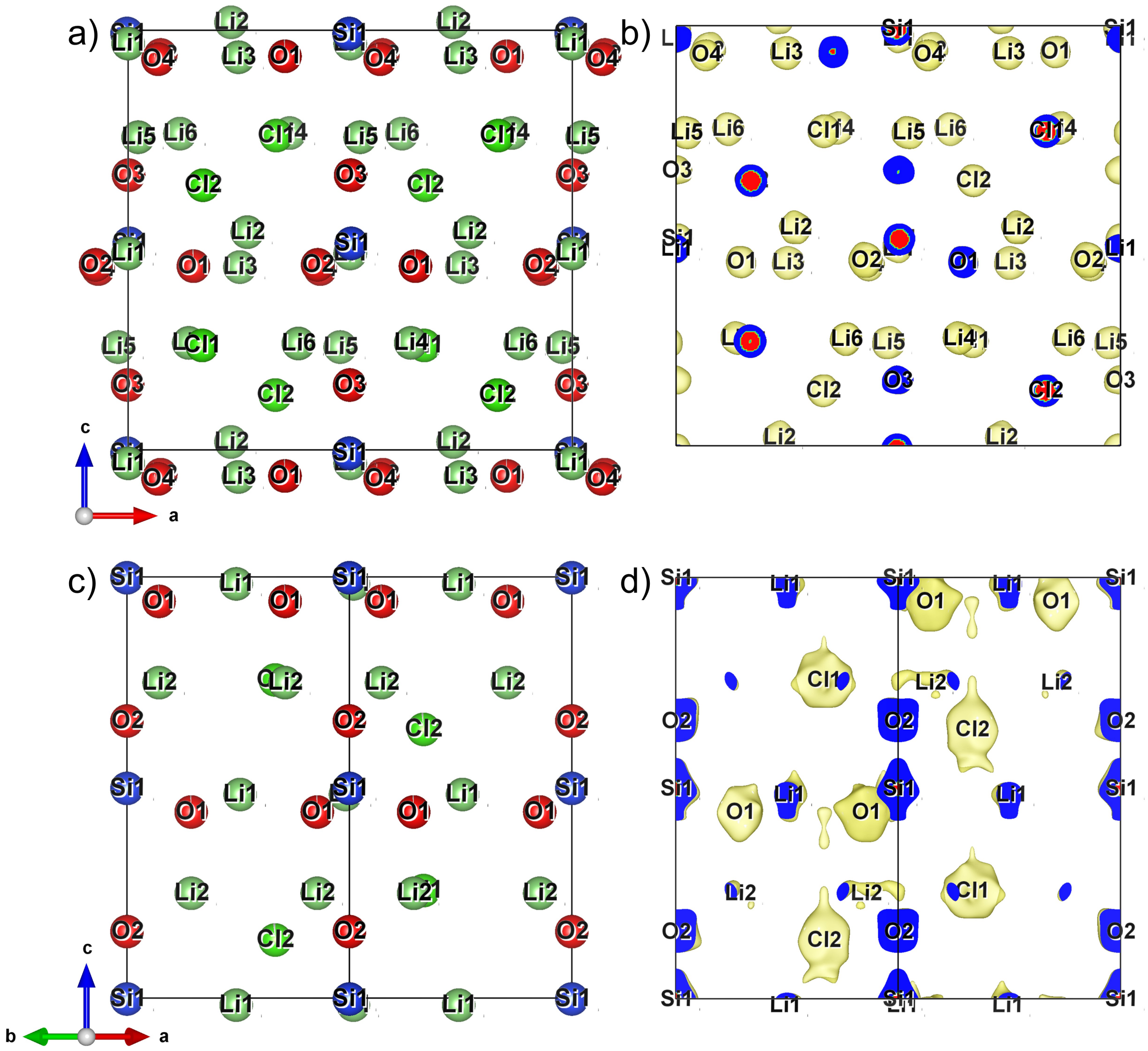

Figure S6: a) RT - $Pna2_1$ model b) MEM analysis showing there are no additional available Li sites c) HT-$P6_3mc$ model without split Li site d) MEM analysis revealing additional electron density around Li2 position indicating displacement onto lower symmetry position

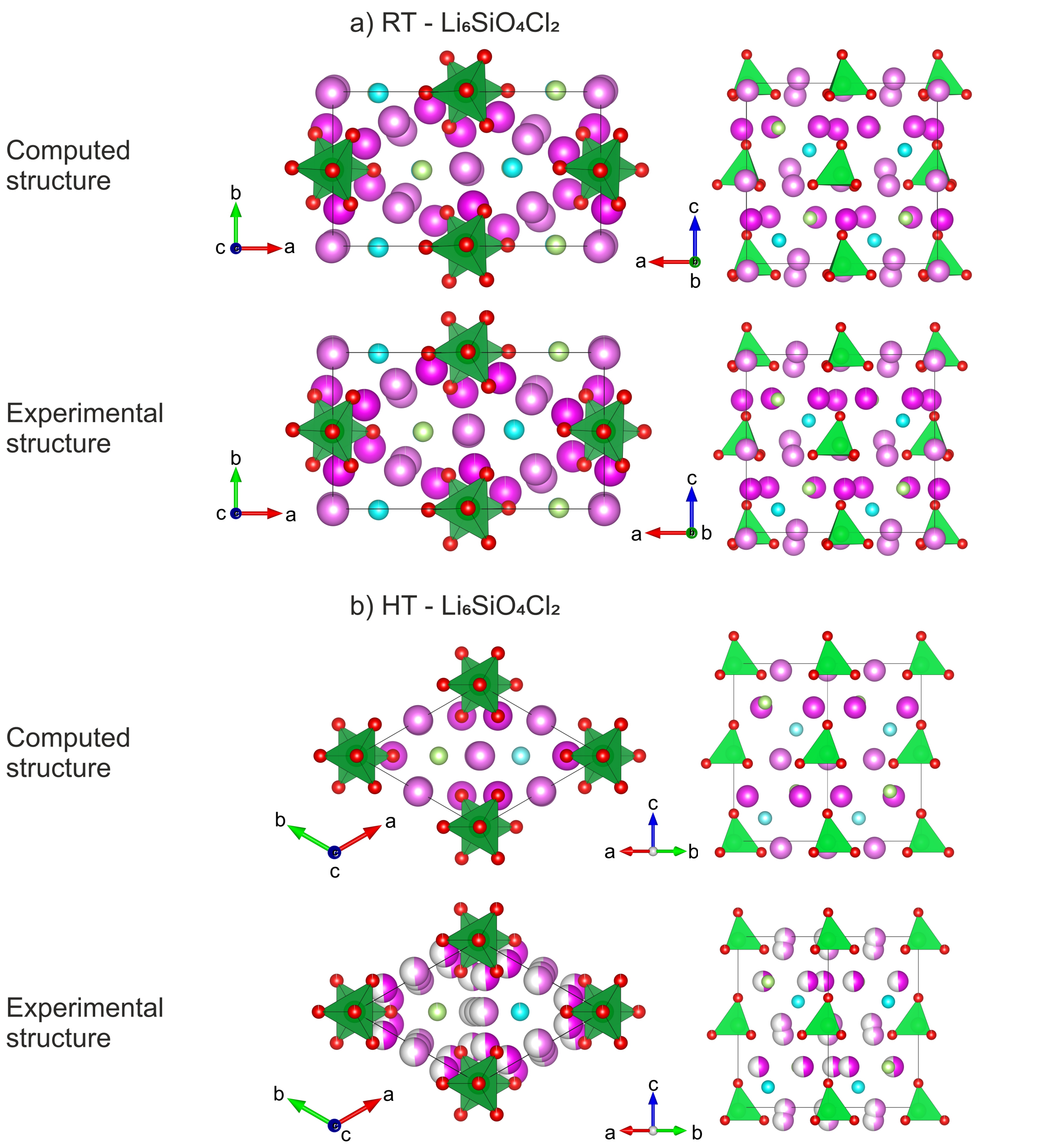

Figure S7: a) Comparison of computed (upper part) and experimental structures (lower part) for RT – $Li_6SiO_4Cl_2$ viewed along the [001] direction (left) and the [010] direction (right) b) Comparison of computed (upper part) to experimental structure (lower part) for HT – $Li_6SiO_4Cl_2$ viewed along the [001] direction (left) and the [110] direction (right) Atom colours: light pink – Li in Li_3SiO_4 layer, dark pink – Li in Li_3Cl layer, light green – A site Cl, blue – B site Cl, green – Si, red – O. In the experimental structure of RT - $Li_6SiO_4Cl_2$, Li is ordered across 4*a* Wyckoff positions. In the experimental structure of HT – $Li_6SiO_4Cl_2$, Li in the Li_3SiO_4 layer (light pink) occupies 6*c* Wyckoff positions, and Li in the Li_3Cl layer (dark pink) occupies 12*d* Wyckoff positions.

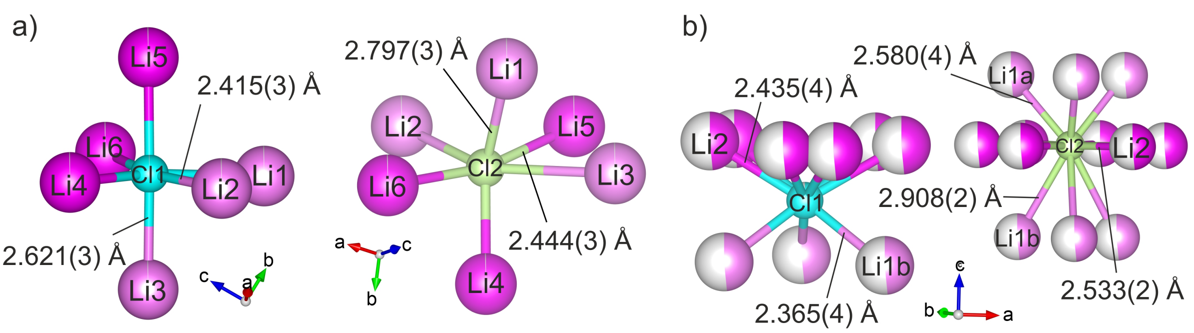

Figure S8: Cl1 and Cl2 polyhedra of a) RT-$Li_6SiO_4Cl_2$ and b) HT-$Li_6SiO_4Cl_2$.

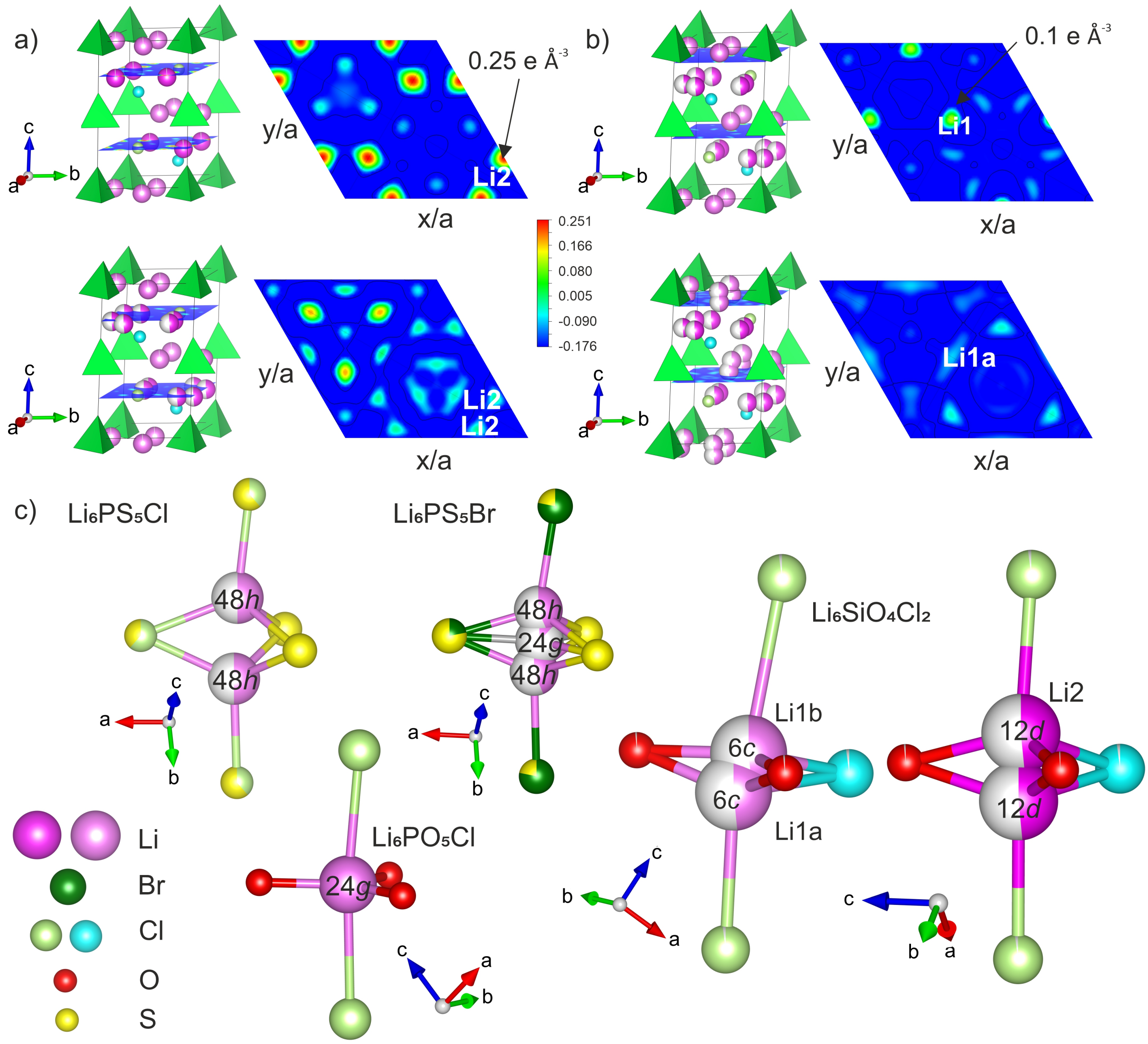

Figure S9: a) upper part: HT – phase with Li2 located on mirror plane (Wyckoff 6*c*) and the electron density observed in the Fourier difference map (FDM), and lower part: HT-phase with Li2 on general position 12*d* and effect on FDM. b) upper part: HT – phase with Li1 fully occupied on Wyckoff position 6*c* and the electron density observed in the Fourier difference map (FDM) and lower part: HT-phase with the splitting of the Li1 site into Li1a and Li1b 6*c* positions with partially occupied sof and effect on FDM. c) Comparison of lithium environments in different argyrodites: Li_6PS_5Cl – lithium split position 48*h*, Li_6PS_5Br lithium position 24*g* and split position 48*h*, Li_6PO_5Cl – lithium position 24*g* and HT-$Li_6SiO_4Cl_2$ -Li2 split position 12*d*, Li1a and Li1b on special position 6*c*

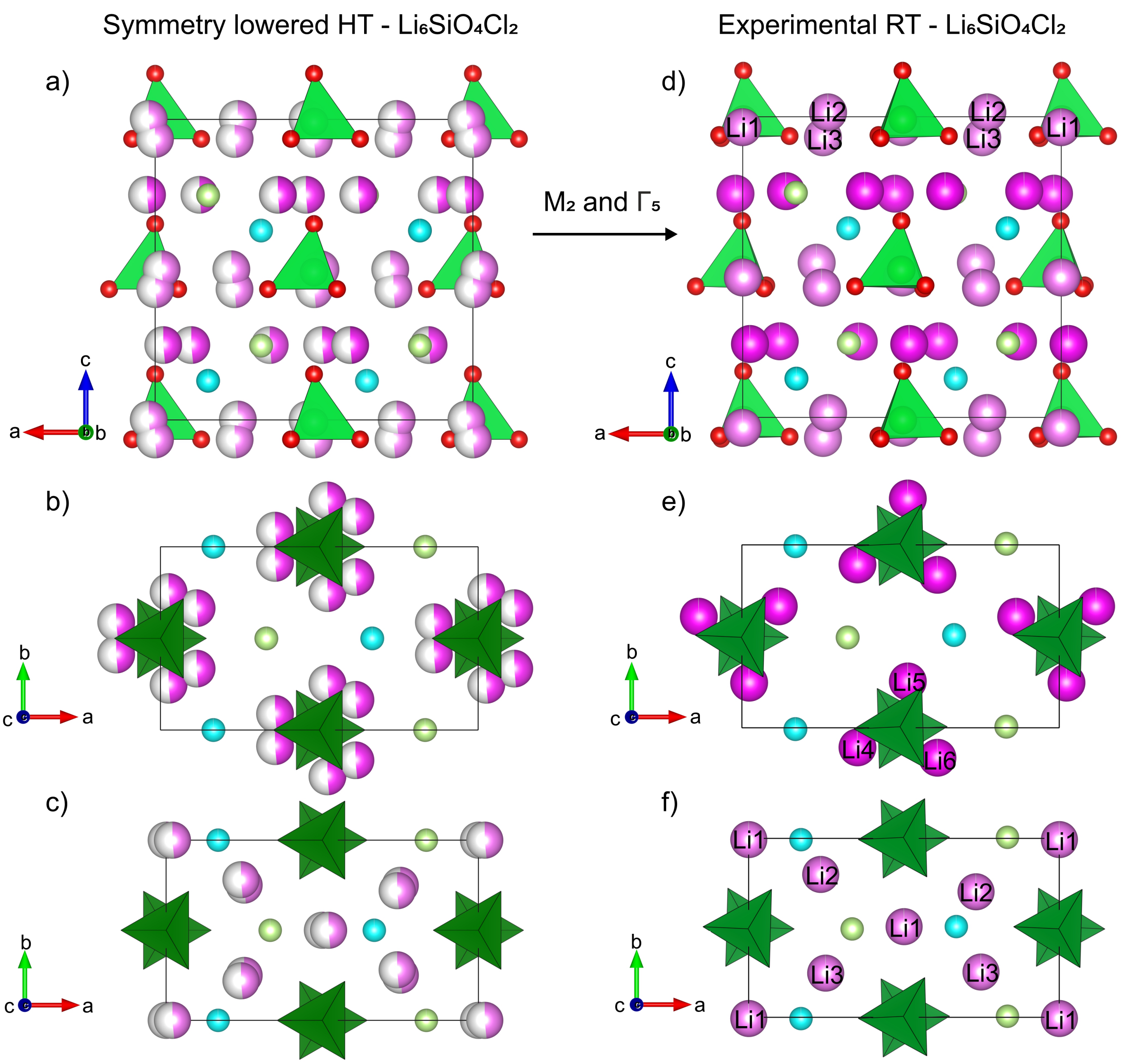

Figure S10: Comparison between a) disordered HT-structure where symmetry is lowered to *Pna*2_1 (obtained from reducing the symmetry of high temperature hexagonal *P*6_3*mc* structure to orthorhombic *Pna*2_1 using ISODISTORT)[1, 2] and b) ordered experimental RT-structure of $Li_6SiO_4Cl_2$, viewed along the [010] axis (Li (Li_3SiO_4 layer): light pink, Li (Li_3Cl layer): dark pink, SiO_4 tetrahedra: light green, Cl: blue and dark green). The (poly)anion network remains unchanged as evidenced by SiO_4 tetrahedra (light green) and Cl positions (blue and green). Investigation of individual layers, c) and d) Li_3Cl layer and e) and f) Li_3SiO_4 layer, shows how activation of the M_2 and Γ_5 occupational modes involving all Li atoms, and Γ_5 displacive modes involving Li1 in the Li_3SiO_4 layer leads to the stabilisation of the ordered RT structure which is observed experimentally.

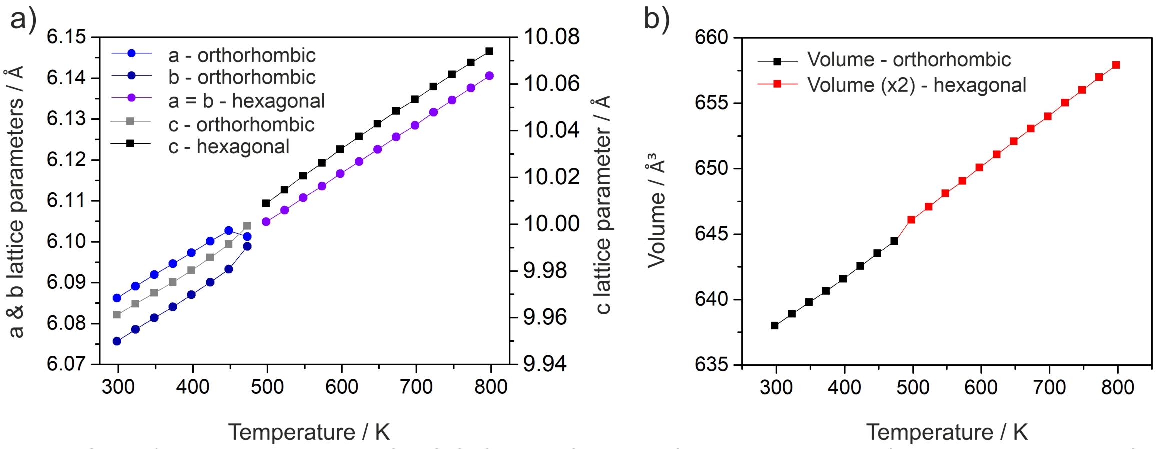

Figure S11: a) Lattice parameters of $Li_6SiO_4Cl_2$ as a function of temperature, and b) unit cell volume as a function of temperature. Error bars are smaller than the data points.

Table S2: Crystal data and structure refinement for $Li_6SiO_4Cl_2$ from single crystal diffraction data collected at 100 K.

Empirical formula	$Li_6O_4SiCl_2$
Formula weight/ g mol^{-1}	204.63
Temperature/K	100.0
Crystal system	Orthorhombic
Space group	$Pna2_1$
a/Å	10.5204(8)
b/Å	6.0756(4)
c/Å	9.9530(7)
Volume/Å^3	636.17(8)
Z	4
ρ_{calc}g/cm^3	2.137
μ/mm^{-1}	1.138
F(000)	392.0
Crystal size/mm^3	0.167 × 0.086 × 0.078
Radiation	MoKα (λ = 0.71073)
2Θ range for data collection/°	7.746 to 52.782
Index ranges	-13 ≤ h ≤ 12, -7 ≤ k ≤ 7, -9 ≤ l ≤ 12
Reflections collected	12264
Independent reflections	1232 [R_{int} = 0.0805, R_{sigma} = 0.0404]
Data/restraints/parameters	1232/79/118
Goodness-of-fit on F^2	1.110
Final R indexes [I>=2σ (I)]	R_1 = 0.0530, wR_2 = 0.1189
Final R indexes [all data]	R_1 = 0.0750, wR_2 = 0.1279
Largest diff. peak/hole / e Å^{-3}	0.64/-0.43
Flack parameter	-0.04(9)

Table S3: Fractional atomic coordinates and equivalent isotropic displacement parameters ($Å^2×10^3$) for $Li_6SiO_4Cl_2$ obtained from refinement against single crystal diffraction data.

Atom	*x*	*y*	*z*	**U(eq)*
Cl1	0.83357(17)	0.4948(4)	0.65663(18)	10.7(5)
Cl2	0.6679(2)	0.9941(4)	0.53638(16)	10.4(5)
Si1	0.5009(2)	0.5028(5)	0.3992(4)	8.3(5)
O1	0.6485(5)	0.4960(8)	0.3427(8)	12.1(11)
O2	0.4305(5)	0.7228(8)	0.3384(7)	9.4(13)
O3	0.4260(5)	0.2826(8)	0.3448(7)	8.8(13)
O4	0.5016(7)	0.5057(11)	0.5636(5)	9.1(14)
Li1	0.6162(14)	0.6620(20)	0.6640(20)	18(3)
Li2	0.5017(15)	1.0090(30)	0.3630(20)	37(4)
Li3	0.3637(16)	0.6020(30)	0.6580(20)	26(4)
Li4	0.2548(13)	0.7540(20)	0.3490(20)	21(4)
Li5	0.4762(16)	0.7540(30)	0.1540(20)	28(4)
Li6	0.2670(20)	0.2030(40)	0.4190(30)	43(5)

*$U_{(eq)}$ is defined as 1/3 of the trace of the orthogonalized U_{ij} tensor (standard deviations in parentheses).

Table S4: Anisotropic displacement parameters ($Å^2×10^3$) for $Li_6SiO_4Cl_2$ obtained from refinement against single crystal diffraction data. The anisotropic displacement factor exponent takes the form: -2π2[h2a*2U11+2hka*b*U12+…].

Atom	U_{11}	U_{22}	U_{33}	U_{23}	U_{13}	U_{12}
Cl1	10.3(10)	7.8(10)	14.1(12)	0.4(13)	1.8(11)	-0.9(9)
Cl2	11.0(9)	8.0(9)	12.2(12)	-0.8(12)	-0.2(9)	-1.6(10)
Si1	8.7(9)	5.5(8)	10.6(10)	-0.1(9)	-0.7(8)	1.1(10)
O1	11(2)	11.6(19)	13(2)	0(2)	1(2)	0(2)
O2	9(2)	7(2)	12(2)	2(2)	0(2)	3.8(18)
O3	7(2)	6(2)	13(2)	-2(2)	0(2)	-2.5(17)
O4	10(2)	8(2)	9(2)	0(2)	1(2)	0(2)
Li1	18(4)	17(4)	18(4)	1(3)	-1(3)	-1(3)
Li2	37(5)	35(5)	38(5)	0(3)	-2(3)	0(3)
Li3	25(4)	27(4)	27(4)	0(3)	0(3)	1(3)
Li4	19(4)	20(4)	24(4)	0(3)	1(3)	1(3)
Li5	28(4)	27(4)	28(5)	0(3)	1(3)	1(3)
Li6	42(6)	42(6)	45(6)	-2(3)	2(3)	1(3)

Table S5: Bond lengths for $Li_6SiO_4Cl_2$ obtained from refinement against single crystal diffraction data.

Atom	Atom	Length/Å	Atom	Atom	Length/Å
Cl1	Li1	2.503(15)	Li2	Cl1[9]	2.689
Cl1	Li2[1]	2.69(2)	Li2	Cl2	2.459
Cl1	Li3[2]	2.468(18)	Li2	O2	1.909
Cl1	Li4[3]	2.615(19)	Li2	O3[8]	1.852
Cl1	Li5[1]	2.480(17)	Li3	Cl2[10]	2.459
Cl1	Li6[4]	2.75(3)	Li3	O1[5]	1.937
Cl2	Li1	2.448(16)	Li3	O4	1.825
Cl2	Li2	2.455(19)	Li3	Cl1[10]	2.47
Cl2	Li3[2]	2.461(18)	Li4	Cl1[6]	2.61
Cl2	Li4[2]	2.564(17)	Li4	Cl2[10]	2.566
Cl2	Li5[5]	2.454(18)	Li4	O1[10]	1.887
Cl2	Li6[2]	2.42(2)	Li4	O2	1.861
Si1	O1	1.652(6)	Li5	Cl2[11]	2.452
Si1	O2	1.643(6)	Li5	O2	1.906
Si1	O3	1.644(6)	Li5	O4[11]	1.831
Si1	O4	1.637(6)	Li5	Cl1[9]	2.479
Li1	Cl2	2.445	Li6	Cl2[10]	2.416
Li1	O3[3]	1.884	Li6	O1[12]	1.895
Li1	O4	1.831	Li6	O3	1.892
Li1	Cl1	2.503	Li6	Cl1[12]	2.744

Symmetry transformations used to generate equivalent atoms:
[1]3/2-X,-1/2+Y,1/2+Z; [2]1/2+X,3/2-Y,+Z; [3]1-X,1-Y,1/2+Z; [4]1/2+X,1/2-Y,+Z; [5]1-X,2-Y,1/2+Z; [6]1-X,1-Y,-1/2+Z; [7]+X,-1+Y,+Z; [8]+X,1+Y,+Z; [9]3/2-X,1/2+Y,-1/2+Z; [10]-1/2+X, 3/2-Y, Z; [11]1-X,2-Y,-1/2+Z; [12]-1/2+X,1/2-Y,+Z;

Table S6: Bond angles for $Li_6SiO_4Cl_2$ obtained from refinement against single crystal diffraction data.

Atom	Atom	Atom	Angle/˚	Atom	Atom	Atom	Angle/˚
Li1	Cl1	Li2[1]	123.5(6)	Cl2	Li1	Cl1	96.6(5)
Li1	Cl1	Li4[2]	83.5(5)	O3[2]	Li1	Cl1	108.5(8)
Li1	Cl1	Li6[3]	88.3(6)	O3[2]	Li1	Cl2	113.6(7)
Li2[1]	Cl1	Li6[3]	146.9(6)	O4	Li1	Cl1	112.1(8)
Li3[4]	Cl1	Li1	73.5(5)	O4	Li1	Cl2	106.8(8)
Li3[4]	Cl1	Li2[1]	83.2(6)	O4	Li1	O3[2]	117.4(8)
Li3[4]	Cl1	Li4[2]	128.0(7)	Cl2	Li2	Cl1[8]	94.4(5)
Li3[4]	Cl1	Li5[1]	118.8(5)	O2	Li2	Cl1[8]	97.1(8)
Li3[4]	Cl1	Li6[3]	118.1(8)	O2	Li2	Cl2	109.7(9)
Li4[2]	Cl1	Li2[1]	71.7(5)	O3[9]	Li2	Cl1[8]	103.2(8)
Li4[2]	Cl1	Li6[3]	106.8(6)	O3[9]	Li2	Cl2	114.1(10)
Li5[1]	Cl1	Li1	167.6(5)	O3[9]	Li2	O2	129.5(9)
Li5[1]	Cl1	Li2[1]	60.4(7)	Cl2[11]	Li3	Cl1[11]	97.2(6)
Li5[1]	Cl1	Li4[2]	87.3(5)	O1[2]	Li3	Cl1[11]	107.7(9)
Li5[1]	Cl1	Li6[3]	86.5(7)	O1[2]	Li3	Cl2[11]	109.8(8)
Li1	Cl2	Li2	103.6(6)	O4	Li3	Cl1[11]	114.7(9)
Li1	Cl2	Li3[4]	74.6(6)	O4	Li3	Cl2[11]	109.5(10)
Li1	Cl2	Li4[4]	88.3(5)	O4	Li3	O1[2]	116.2(10)
Li1	Cl2	Li5[5]	97.4(6)	Cl2[11]	Li4	Cl1[6]	93.7(5)
Li2	Cl2	Li3[4]	161.8(7)	O1[11]	Li4	Cl1[6]	103.2(8)
Li2	Cl2	Li4[4]	76.5(6)	O1[11]	Li4	Cl2[11]	106.7(7)
Li3[4]	Cl2	Li4[4]	85.3(6)	O2	Li4	Cl1[6]	104.5(8)
Li5[5]	Cl2	Li2	82.6(7)	O2	Li4	Cl2[11]	109.7(8)
Li5[5]	Cl2	Li3[4]	115.5(7)	O2	Li4	O1[11]	132.0(8)
Li5[5]	Cl2	Li4[4]	159.1(7)	Cl2[10]	Li5	Cl1[8]	97.7(6)
Li6[4]	Cl2	Li1	167.3(6)	O2	Li5	Cl1[8]	104.6(9)
Li6[4]	Cl2	Li2	86.6(7)	O2	Li5	Cl2[10]	111.5(8)
Li6[4]	Cl2	Li3[4]	93.4(7)	O4[6]	Li5	Cl1[8]	114.2(8)
Li6[4]	Cl2	Li4[4]	86.7(8)	O4[6]	Li5	Cl2[10]	112.4(10)
Li6[4]	Cl2	Li5[5]	91.3(7)	O4[6]	Li5	O2	114.9(9)
O2	Si1	O1	108.6(4)	Cl2[11]	Li6	Cl1[12]	91.4(9)
O2	Si1	O3	108.9(3)	O1[12]	Li6	Cl1[12]	103.5(10)
O3	Si1	O1	108.6(3)	O1[12]	Li6	Cl2[11]	113.3(10)
O4	Si1	O1	109.6(4)	O3	Li6	Cl1[12]	102.8(10)
O4	Si1	O2	111.2(4)	O3	Li6	Cl2[11]	112.0(10)
O4	Si1	O3	109.9(4)	O3	Li6	O1[12]	126.2(14)

Symmetry transformations used to generate equivalent atoms:
[1]3/2-X,-1/2+Y,1/2+Z; [2]1-X,1-Y,1/2+Z; [3]1/2+X,1/2-Y,+Z; [4]1/2+X,3/2-Y,+Z; [5]1-X,2-Y,1/2+Z; [6]1-X,1-Y,-1/2+Z; [7]+X,-1+Y,+Z; [8]3/2-X,1/2+Y,-1/2+Z; [9]+X,1+Y,+Z; [10]1-X,2-Y,-1/2+Z; [11]-1/2+X,3/2-Y,+Z; [12]-1/2+X,1/2-Y,+Z

Table S7: Structure refinement against powder diffraction data of room temperature (293 K) and high temperature (548 K) phase of $Li_6SiO_4Cl_2$.

**Phase**	**Room temperature**	**High temperature**
Empirical Formula	$Li_{5.957(2)}Si_{1.00}O_{3.986(2)}Cl_{1.985(1)}$	$Li_{5.824(1)}Si_{1.00}O_{3.934(6)}Cl_{1.956(4)}$
Formula Weight (g mol^{-1})	204.64	204.64
Space group	$Pna2_1$	$P6_3mc$
Z	4	2
Density (g cm^{-3})	2.130	2.097
Temperature (K)	293.15	548.15
Wavelength (Å)	0.826552	0.826596
d – spacing range (Å)	0.63246 - 5.9329	0.71678 - 5.9329
No. of reflections	1390	208
No. of refined parameters	121	84
a (Å)	10.543150(5)	6.110780(11)
b (Å)	6.076855(3)	/
c (Å)	9.960249(5)	10.02065(2)
Volume (Å)	638.144(1)	324.055(1)
R_p	3.11	1.95
R_{wp}	4.27	2.80
R_{exp}	1.64	0.57
χ^2	2.60	4.90

Table S8: Atomic positions, isotropic atomic displacement parameters (U_{iso}, Å^2×10^3), and site occupancy factors for RT – $Li_6SiO_4Cl_2$ and HT - $Li_6SiO_4Cl_2$ obtained from refinement against powder diffraction data. Anisotropic displacement parameters (Å^2×10^3) are refined for Cl positions and given in the table.

Site	Wyckoff position	x	y	z	sof	U_{iso} (Å^2×10^3)
			**RT – $Li_6SiO_4Cl_2$**			
Li1	4*a*	0.0005(2)	-0.0067(8)	-0.0067(8)	0.993(2)	22.4(8)
Li2	4*a*	0.2679(3)	0.2985(5)	0.5146(3)	0.993(2)	11(1)
Li3	4*a*	0.7519(3)	0.7531(4)	0.4316(4)	0.993(2)	14.2(9)
Li4	4*a*	0.3642(3)	0.8928(4)	0.7525(5)	0.993(2)	17(1)
Li5	4*a*	0.0228(3)	0.2465(4)	0.7442(4)	0.993(2)	13(1)
Li6	4*a*	0.6162(3)	0.8363(5)	0.7531(4)	0.993(2)	12(1)
Si	4*a*	0.50232(4)	0.99692(17)	0.49074(6)	1.000	7.3(7)
O1	4*a*	0.50179(12)	0.9947(3)	0.65312(7)	0.993(1)	9.6(3)
O2	4*a*	0.42622(10)	0.21544(17)	0.43910(13)	1.000(3)	12.4(5)
O3	4*a*	0.43299(11)	0.77773(17)	0.42900(13)	0.9934(8)	8.7(5)
O4	4*a*	0.64698(7)	0.00621(19)	0.43587(14)	1.000(3)	9.5(3)
Cl1	4*a*	0.66862(4)	0.50718(9)	0.62894(3)	0.9939(6)	13.1(2)
Cl2	4*a*	0.33328(4)	0.49395(9)	0.74719(3)	0.9915(6)	16.2(2)
**Atom**	**U_{11}**	**U_{22}**	**U_{33}**	**U_{23}**	**U_{13}**	**U_{12}**
Cl1	12.6(2)	12.1(2)	14.5(2)	-1.8(3)	-0.1(2)	-0.6(4)
Cl2	14.2(2)	13.5(2)	20.9(2)	2.4(3)	-0.2(4)	-1.3(4)
			**HT – $Li_6SiO_4Cl_2$**			
Li1a	6*c*	0.5003(6)	0.4997(6)	-0.0759(11)	0.485(2)	19(2)
Li1b	6*c*	0.4793(5)	0.5207(5)	-0.5094(9)	0.485(2)	14(2)
Li2	12*d*	0.7725(6)	0.0512(5)	0.7383(4)	0.485(2)	33(1)
Si	2*a*	0	0	-0.0194 (1)	1.000	9.88(12)
O1	6*c*	0.14479(6)	0.85521(6)	0.42440(16)	0.982(3)	14.3(3)
O2	2*a*	0	0	0.64116(18)	0.988(2)	15.2(5)
Cl1	2*b*	1/3	2/3	0.11937(4)	0.977(2)	23.5(7)
Cl2	2*b*	1/3	2/3	0.73651(8)	0.979(2)	29.7(8)
**Atom**	**U_{11}**	**U_{22}**	**U_{33}**	**U_{23}**	**U_{13}**	**U_{12}**
Cl1	21.3(8)	21.7(6)	27.4(7)	10.0(10)	-1.2(4)	-0.2(5)
Cl2	24.6(9)	24.6(7)	39.8(9)	10.7(10)	-5.6(9)	5.0(10)

Table S9: Selected interatomic distances, angles and bond valence sums for HT and RT – $Li_6SiO_4Cl_2$ obtained from refinement against powder diffraction data. Bond valence sums (BVS) for each atom were calculated using the formalism defined by Brese and O'Keeffe,[3] where the total valence of an atom *i* can be expressed as the sum of the valence contributions from the individual bonds to neighbouring atoms *j*, and is determined from their lengths d_{ij} through $V_i = \Sigma v_{ij} = \Sigma \exp[(R_{ij}-d_{ij})/b]$, where *b* is a constant, and R_{ij} is the atom-specific bond valence parameter.

Central Atom	Distances/ Å		Angles/ °		Coordination	BVS
			HT – $Li_6SiO_4Cl_2$			
Cl1	Li1b	2.366(6)	Li1b Cl1 Li1b	93.1(3)	6	/
	Li2	2.434(4)	Li1b Cl1 Li2	94.25(14)		
				166.89(7)		
				75.68(18)		
			Li2 Cl1 Li2	98.09(12)		
				76.60(15)		
				25.58(12)		
				114.52(17)		
Cl2	Li1a	2.580(9)	Li1a Cl2 Li1a	72.8(3)	12	/
	Li1b	2.908(8)	Li1a Cl2 Li1b	104.7(2)		
				143.06(12)		
	Li2	2.533(3)	Li1a Cl2 Li2	128.56(19)		
				56.28(16)		
				85.23(9)		
			Li1b Cl2 Li1b	65.11(13)		
				119.56(15)		
				86.86(9)		
			Li2 Cl2 Li2	46.89(19)		
				119.995(8)		
				166.87(19)		
				73.11(19)		
Li1a	O1	1.889(2)	O1 Li1a O1	132.3(3)	4	0.942(10)
	Cl1	2.636(9)	O1 Li1a Cl1	105.6(3)		
	Cl2	2.580(9)	O1 Li1a Cl2	106.1(3)		
			Cl1 Li1a Cl2	94.69(18)		
Li1b	O1	1.931(4)	O1 Li1b O1	127.1(4)	4	0.928(12)
	Cl1	2.366(6)	O1 Li1b Cl1	115.16(14)		
	Cl2	2.908(8)	O1 Li1b Cl2	98.1(2)		
			Cl1 Li1b Cl2	89.1(3)		
Li2	O1	1.942(4)	O1 Li2 O2	116.7(2)	4	1.062(11)
	O2	1.847(4)	O1 Li2 Cl1	112.56(17)		
	Cl1	2.434(4)	O1 Li2 Cl2	106.19(15)		
	Cl2	2.533(3)	O2 Li2 Cl1	110.55(17)		
			O2 Li2 Cl2	112.16(15)		
			Cl1 Li2 Cl2	96.91(13)		
Si	O1	1.632(1)	O1 Si O1	108.77(8)	4	3.973(14)
	O2	1.609(2)	O1 Si O2	110.16(7)		
			RT – $Li_6SiO_4Cl_2$			
Cl1	Li1	2.417(3)	Li1 Cl1 Li2	88.56(13)	6	/
	Li2	2.417(3)	Li1 Cl1 Li3	76.19(12)		
	Li3	2.620(3)	Li1 Cl1 Li4	163.25(13)		
	Li4	2.477(3)	Li1 Cl1 Li5	80.33(13)		
	Li5	2.476(3)	Li1 Cl1 Li6	101.86(14)		
	Li6	2.415(3)	Li2 Cl1 Li3	86.55(10)		
			Li2 Cl1 Li4	93.57(11)		
			Li2 Cl1 Li5	90.51(10)		
			Li2 Cl1 Li6	167.56(11)		
			Li3 Cl1 Li4	87.34(11)		
			Li3 Cl1 Li5	156.40(11)		
			Li3 Cl1 Li6	89.35(11)		
			Li4 Cl1 Li5	116.22(12)		
			Li4 Cl1 Li6	74.50(10)		
Cl2	Li1	2.798(3)	Li1 Cl2 Li2	147.25(12)	6	/
	Li2	2.693(3)	Li1 Cl2 Li3	71.18(11)		
	Li3	2.537(3)	Li1 Cl2 Li4	82.64(14)		
	Li4	2.446(3)	Li1 Cl2 Li5	61.13(12)		
	Li5	2.476(3)	Li1 Cl2 Li6	122.99(12)		
	Li6	2.511(3)	Li2 Cl2 Li3	105.74(10)		
			Li2 Cl2 Li4	119.33(13)		
			Li2 Cl2 Li5	86.33(11)		
			Li2 Cl2 Li6	88.18(12)		

			Li3 Cl2 Li4	128.16(12)		
			Li3 Cl2 Li4	128.16(12)		
			Li3 Cl2 Li5	86.87(10)		
			Li3 Cl2 Li6	84.45(11)		
			Li4 Cl2 Li5	118.52(9)		
Li1	O2	1.875(5)	Cl1 Li1 Cl2	93.30(8)	4	0.985(11)
	O3	1.900(5)	Cl1 Li1 O2	93.51(14)		
	Cl1	2.417(3)	Cl1 Li1 O3	100.24(14)		
	Cl2	2.798(3)	Cl2 Li1 O2	111.51(18)		
			Cl2 Li1 O3	115.40(19)		
			O2 Li1 O3	129.92(14)		
Li2	O2	1.899(4)	Cl1 Li2 Cl2	92.48(10)	4	0.986(8)
	O3	1.910(3)	Cl1 Li2 O1	104.37(12)		
	Cl1	2.417(3)	Cl1 Li2 O3	103.50(13)		
	Cl2	2.693(3)	Cl2 Li2 O1	112.35(14)		
			Cl2 Li2 O3	111.31(13)		
			O1 Li2 O3	126.24(16)		
Li3	O3	1.918(3)	Cl1 Li3 Cl2	94.94(8)	4	0.939(8)
	O4	1.895(3)	Cl1 Li3 O1	106.92(13)		
	Cl1	2.620(3)	Cl1 Li3 O2	106.59(12)		
	Cl2	2.537(3)	Cl2 Li3 O1	104.42(13)		
			Cl2 Li3 O2	106.67(12)		
			O1 Li3 O2	131.38(15)		
Li4	O1	1.862(4)	Cl1 Li4 Cl2	96.97(10)	4	1.08(1)
	O4	1.930(5)	Cl1 Li4 O1	109.17(16)		
	Cl1	2.477(3)	Cl1 Li4 O4	114.99(15)		
	Cl2	2.446(3)	Cl2 Li4 O1	109.93(12)		
			Cl2 Li4 O4	107.50(17)		
			O1 Li4 O4	116.44(15)		
Li5	O1	1.829(3)	Cl1 Li5 Cl2	98.04(9)	4	1.12(1)
	O3	1.908(4)	Cl1 Li5 O2	104.10(15)		
	Cl1	2.461(3)	Cl1 Li5 O4	114.56(15)		
	Cl2	2.476(3)	Cl2 Li5 O2	110.99(13)		
			Cl2 Li5 O4	112.54(17)		
			O2 Li5 O4	115.09(14)		
Li6	O1	1.837(4)	Cl1 Li6 Cl2	96.83(11)	4	1.10(1)
	O3	1.931(3)	Cl1 Li6 O3	107.62(15)		
	Cl1	2.415(3)	Cl1 Li6 O4	111.80(14)		
	Cl2	2.511(3)	Cl2 Li6 O3	114.10(13)		
			Cl2 Li6 O4	107.71(17)		
			O3 Li6 O4	117.00(16)		
Si	O1	1.6174(9)	O1 Si O2	108.64(8)	4	3.958(12)
	O2	1.6345(14)	O1 Si O3	111.53(8)		
	O3	1.6391(14)	O1 Si O4	109.92(7)		
	O4	1.6211(10)	O2 Si O3	108.86(6)		
			O2 Si O4	109.11(8)		
			O3 Si O4	108.75(7)		

3. Ionic conductivity study

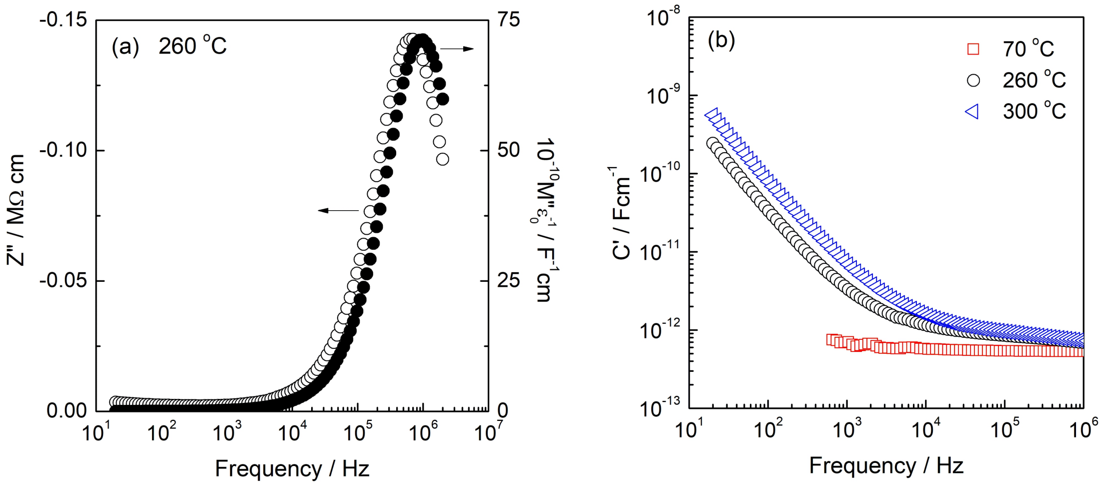

Figure S12: a) Combined Z"/M" spectra and b) Capacitance vs frequency at different temperatures. The overlap of the Z" peak and M' peak in a) indicates the sample is homogeneous. The high-frequency plateau in b) has a value of ~0.6 pF cm^{-1} and thus represents the bulk response.

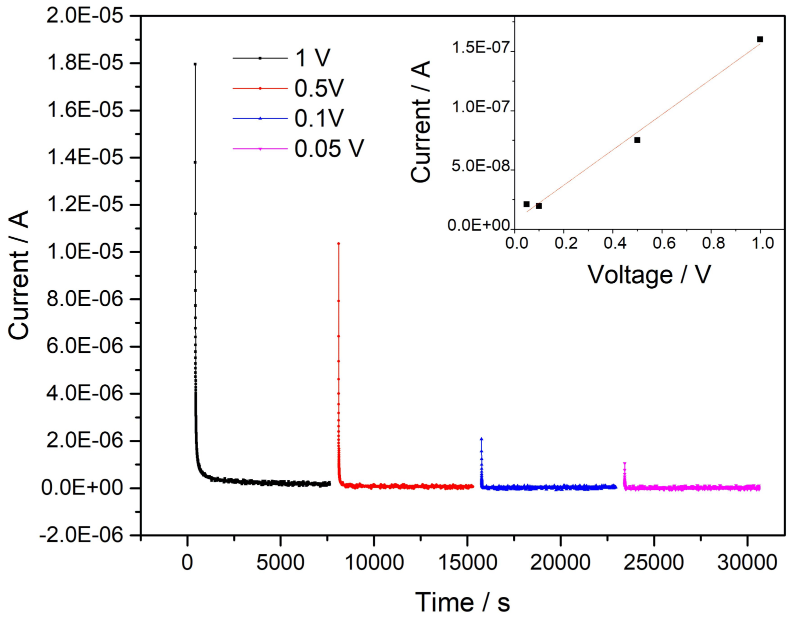

Figure S13: DC polarisation data measured at 300 °C for 0.05V, 0.1V, 0.5V and 1V. The electronic conductivity was extracted by plotting the current vs the applied voltage as shown in the inset and found to be 2.92×10^{-8} S cm^{-1} at 300 °C which is a contribution of less than 0.5 % to the overall conductivity.

References

1. Stokes, H. T.; Hatch, D. M.; Campbell, B. J.; Tanner, D. E. ISODISPLACE: a web-based tool for exploring structural distortions. *J. Appl.Crystallogr.* **2006,** 39, 607-614.
2. Stokes, H. T.; Hatch, D. M.; Campbell, B. J., ISODISTORT, ISOTROPY Software Suite, Iso. Byu. Edu.
3. Brese, N. E.; O'Keeffe, M. Bond-Valence Parametrers for Solids. *Acta Cryst. B* **1991,** 47, 192-197
